# Feasibility of a family-oriented mHealth intervention for Chinese Americans with type 2 diabetes: A pilot randomized control trial

**DOI:** 10.1371/journal.pone.0299799

**Published:** 2024-03-11

**Authors:** Lu Hu, Yun Shi, Judith Wylie-Rosett, Mary Ann Sevick, Xinyi Xu, Ricki Lieu, Chan Wang, Huilin Li, Han Bao, Yulin Jiang, Ziqiang Zhu, Ming-Chin Yeh, Nadia Islam

**Affiliations:** 1 Institute for Excellence in Health Equity, Center for Healthful Behavior Change, NYU Langone Health, New York, NY, United States of America; 2 Department of Population Health, NYU Grossman School of Medicine, NYU Langone Health, New York, NY, United States of America; 3 Department of Epidemiology & Population Health, Albert Einstein College of Medicine, New York, NY, United States of America; 4 Department of Medicine, Albert Einstein College of Medicine, New York, NY, United States of America; 5 Department of Medicine, NYU Grossman School of Medicine, NYU Langone Health, New York, NY, United States of America; 6 Jacobi Medical Center, New York, NY, United States of America; 7 Wellsure Medical Practice, New York, NY, United States of America; 8 School of Urban Public Health, Hunter College, City University of New York, New York, NY, United States of America; Medical College of Wisconsin, UNITED STATES

## Abstract

**Objectives:**

To test the feasibility, acceptability, and potential efficacy of a mHealth intervention tailored for Chinese immigrant families with type 2 diabetes (T2D).

**Methods:**

We conducted a pilot randomized controlled trial (RCT) with baseline, 3-, and 6-month measurements. Participating dyads, T2D patients and families/friends from NYC, were randomized into the intervention group (n = 11) or the wait-list control group (n = 12). Intervention includes 24 videos covering T2D self-management, behavioral techniques, and family-oriented sessions. Feasibility and acceptability were measured respectively by the retention rate and video watch rate, and a satisfaction survey. Patients’ HbA1c, weight, and self-management were also assessed to test potential efficacy.

**Results:**

Most T2D patients (n = 23; mean age 56.2±9.4 years; 52.2% male) and families/friends (n = 23, mean age 54.6±11.2 years; 52.2% female) had high school education or less (69.6% and 69.6%), annual household income < $25,000 (65.2% and 52.2%), and limited English proficiency (95.7% and 95.7%). The retention rates were not significantly different between the intervention and the control groups for both the patients (90.91% vs 83.3%, p = 0.589); and their families/friends (3-month: 90.9% vs 75%, p = 0.313; 6-month: 90.9% vs 83.3%, p = 0.589). The mean video watch rate was 76.8% (7%). T2D patients and families/friends rated satisfaction as 9.4 and 10 out of 10, respectively. Despite no between-group differences, the intervention group had significantly lower HbA1c (p = 0.014) and better self-management (p = 0.009), and lost 12 lbs. on average at 6 months (p = 0.079), compared to their baseline levels.

**Conclusions:**

A culturally-tailored, family-based mHealth intervention is feasible and acceptable among low-income, limited English-proficient Chinese families with T2D in NYC. Significant changes in HbA1c and self-management within the intervention group indicate this intervention may have potential efficacy. Given the small sample size of this study, a future RCT with adequate power is needed to test efficacy.

## Introduction

Chinese Americans are the largest Asian subgroup in the United States, with a population of 5.2 million. [1] Among those aged 45–64, up to 33.8% have type 2 diabetes (T2D), and 13.3% have prediabetes [2]. Compared to non-Hispanic Whites, Chinese Americans have a higher prevalence of T2D [3–7], demonstrated worse self-management and glycemic control [8–12], and had a greater risk of developing renal complications [13]. However, T2D prevention and management in this population are particularly challenging due to various factors such as limited English proficiency, communication barriers with healthcare providers, and limited access to healthcare services [14–16]. Moreover, the lack of culturally and linguistically concordant health providers has further contributed to the disparities in T2D care and health outcomes in this population [14–16]. Considering the substantial and growing number of Chinese Americans in the United States [17–19] and the high prevalence of T2D in this population, evidence-based diabetes management interventions are urgently needed [20].

Diabetes management is largely influenced by the social and environmental contexts in which patients live and manage their diabetes [21,22]. Increasing evidence suggests that family member’s education and empowerment are critical for diabetes management [21,23,24]. Chinese Americans may particularly benefit from interventions involving their families and friends due to the strong emphasis on family ties in Asian culture [23,25,26]. Families are often willing to offer support and assistance when a member suffers from a disease and are ideally positioned to provide long-term support that lasts beyond professional interventions [26–28]. Yet, many family members are frustrated because they lack the knowledge and skills to better support and help their loved ones [21,29]. As most interventions reported in the literature failed to involve family members [23,30], we developed a diabetes self-management intervention involving both patients and family members to fill this gap.

A mobile health (mHealth) short message service (SMS) was selected for delivering the intervention given that our pilot data showed most Chinese Americans had smartphones and frequently used text messaging [31]. Specifically, our previous survey revealed that 91% of Chinese Americans own a smart device (smartphone or tablet), with 71% using text messaging applications such as WeChat [31,32]. Chinese Americans are especially likely to benefit from mHealth since they often work long hours and have difficulty attending in-person interventions [15,27,28]. In addition, they are quite familiar with SMS because they use it to connect with family and friends in the US and China [31]. The majority of participants in our recent mixed-method study of 101 Chinese American patients with T2D endorsed the idea of SMS-based interventions and reported that their family members would also be interested in receiving such an intervention [31]. Hence, we developed a mHealth intervention named FAMILY for both patients and their families. In the current study, we aim to 1) assess the feasibility and acceptability of the FAMILY intervention in Chinese Americans with T2D and their family members; and 2) establish proof-of-concept regarding the potential efficacy of the FAMILY intervention for improving glycemic control, body weight, and psychosocial and behavioral factors.

## Materials and methods

### Design

This study is a 6-month pilot randomized controlled trial (RCT), with measurements at baseline, 3, and 6 months. Participating dyads, patients with T2D and their families/friends, were randomly assigned to either the FAMILY intervention group (n = 11) or the wait-list control group (n = 12) via a computer-generated randomization scheme. The intervention group received a 12-week diabetes self-management educational intervention called the FAMILY intervention, which is described in detail in a later section of this paper. For participants in the control group, patients continued to receive the standard T2D care from their healthcare providers during the study, and as an incentive, both control patients and their families/friends received the FAMILY videos at the end of the study. Participants in both the intervention and control groups were thoroughly briefed about the study procedures, including the information that the control group would receive the same videos at the study’s conclusion. All participants gave informed consent before their involvement in the study. The study protocol was approved by the NYU Grossman School of Medicine Institutional Review Board (protocol s19-01275; S1, S3 and S4 Files) and was registered at ClinicalTrials.gov (NCT04108299; S2 File).

### Participants and recruitment

This study required dyadic participation of patients with T2D and their families/friends. The inclusion criteria for patients with T2D include: 1) self-identified as Chinese immigrant or Chinese American; 2) been between the ages of 18 and 70; 3) been able to speak and understand Mandarin (because our videos are in Mandarin); 4) had a medical diagnosis of T2D; 5) had baseline HbA1c ≥ 7%; 6) been currently using WeChat (a popular text messaging application among Chinese Americans) or text messages; 7) been willing to receive WeChat or text messages regarding T2D management; 8) expressed strong interest and confidence in finishing watching 2 diabetes videos each week for a total of 12 weeks; 9) been motivated to make lifestyle changes to control their diabetes; and 10) had a family member or friend be willing to participate in the study to learn about T2D to better support them.

The inclusion criteria for family members include: 1) self-identified as Chinese immigrant or Chinese American; 2) been between 18–70 years old; 3) been able to speak and understand Mandarin; 4) been currently using WeChat or text messages; 5) been willing to receive WeChat or text messages regarding T2D management and learn how to better support the patient with T2D; 6) expressed strong interest and confidence in finishing watching 2 diabetes videos each week for a total of 12 weeks; and 7) been motivated to support their families/friends to make lifestyle changes to control their diabetes.

The participants were recruited through direct referrals from healthcare providers and NYU Langone Health (NYULH) electronic medical record system (EPIC). Before enrolling participants, the study staff conducted a quick phone screener to determine eligibility. Upon confirming eligibility, potential participants were informed about the purpose of the study, as well as the requirements for active participation, including verbal consent, interviews, and potential benefits and risks. Due to COVID-19, an IRB-approved audio-recorded verbal consent was collected from each participant before any formal study procedures. The study CHW read the IRB-approved verbal script to participants and recorded this consent process via video-conferencing for documentation purposes.

### FAMILY intervention

The FAMILY intervention was adapted from our prior successfully tested CARE intervention. CARE intervention is a culturally and linguistically tailored diabetes self-management education and support program developed for underserved Chinese Americans with T2D. In a feasibility study, we found high feasibility, acceptability, and potential efficacy for reducing HbA1c in this population [33]. However, the CARE intervention was tested solely on patients, without involving family and friends. Thus, we adapted the patient-focused CARE intervention into a family-centered FAMILY intervention guided by the Individual and Family Self-Management Theory [22]. Based on this theory, the FAMILY intervention covers context, process, and outcome factors that may influence patient and family self-management behaviors.

FAMILY is a 12-week intervention program offering diabetes self-management education (DSME) videos that can be accessed at any time and place convenient for the participants. Participants and their families/friends in the FAMILY intervention group received the same 2 videos every week for 12 weeks, with each video lasting approximately 5–10 minutes. These videos provide information on T2D, diabetes self-management at home, behavioral techniques, and family-oriented sessions. The videos were shared through WeChat, the predominant social media platform among Chinese Americans [32]. To ensure engagement, participants who had missed three consecutive videos were contacted to identify barriers to watching and to remind them to watch the videos.

Besides videos, patients and their families/friends also received separate biweekly phone calls from our trained community health workers (CHWs). For patient participants, our CHWs will guide them to set goals for their diabetes management during the initial biweekly phone call, with subsequent calls monitoring progress towards these goals and addressing any questions/concerns they raise. For families/friends participants, our study CHWs shared the goals that patients identified and encouraged families/friends participants to support patients in achieving these goals (e.g., offering to take a walk with the patient after dinner to help the patient achieve the walking goal for that week).

### Measurements

#### Primary outcomes

The primary outcomes of this study are the feasibility and acceptability of the FAMILY intervention. The feasibility of the intervention was determined by calculating the percentage of videos sent that were viewed. In addition, we assessed the feasibility by calculating whether we met our goal of recruiting 30 dyads of patients with T2D and their families/friends and achieving an 80% retention rate. Acceptability was assessed using a satisfaction questionnaire adapted from a prior SMS intervention study [34].

#### Secondary outcomes

Patients’ secondary outcomes include HbA1c, self-reported weight, dietary intake, physical activity, self-efficacy, diabetes self-management behaviors, diabetes distress, and support. Patients’ HbA1c results were abstracted from NYULH Epic or the medical record of their healthcare providers, and other secondary outcomes were measured using self-report questionnaires. The adapted Mediterranean Dietary Screener [35] was used to estimate dietary intake with a higher score indicating more food consumptions. It contains 6 items and asks respondents about the types of food they consumed in the past 30 days, including fruits, vegetables, refined grains, whole wheat, sugary drinks, and starchy foods. The International Physical Activity Questionnaire (IPAQ) short version [36] was used to assess how much time the respondents spend over the past 7 days doing vigorous, moderate, and mild-intensity physical activities. A higher IPAQ scores indicate higher levels of physical activity. The well-validated Stanford Diabetes Self-Efficacy Scale [37,38] was used to measure patients’ confidence to manage T2D. This instrument contains 8 items and asks respondents to rate their confidence level in performing specific self-management behaviors, using a 10-point Likert scale ranging from 1 (not at all confident) to 10 (totally confident); a higher score indicates greater self-efficacy. The adapted Summary of Diabetes Self-Care Activities (SDSCA) questionnaire [39] was used to assess patients’ adherence to diabetes self-management behaviors. This scale consists of 13 items and asks respondents to describe their diabetes self-care activities over the past 7 days. Higher scores of SDSCA indicate greater adherence to self-management behaviors. The Diabetes Distress Scale (DDS) [40] was used to measure patients’ distress levels. This scale consists of 17 items and asks respondents to describe the diabetes-related distress experienced within the past month, including emotional distress, physician-related distress, regimen-related distress, and interpersonal distress. The items are scored on a 6- point Likert scale, ranging from 1 (not a problem) to 6 (a very serious problem), with higher scores indicating higher levels of diabetes distress. The Patient Reported Outcomes Measurement Information System (PROMIS) Emotional Support Short Form v2.0 [41] was used to capture the availability of others with whom they could talk and feel appreciated. The participants were also asked about diabetes-specific support, such as medication taking, healthy diet, physical activity, blood sugar monitoring, stress management, and diabetes management. Higher scores of these two questionnaires indicate better support. We also collected the following information from families/friends using the questionnaires mentioned above: self-reported weight, dietary intake, physical activity, and support. In addition, we tested families/friends’ diabetes knowledge. All the secondary outcomes were measured at baseline, 3 months, and 6 months.

### Data analysis

We employed the “intent-to-treat” (ITT) approach in analyses. We performed a detailed descriptive analysis of all the data collected in the study. These preliminary descriptive statistics were used to 1) check the accuracy and completeness of inputted data, 2) describe the univariate distribution of each variable at baseline, and 3) examine the associations between variables. We also explored features of the data (e.g., amount and pattern of missing data, outliers, excess zeros, departures from distributional assumptions) to determine whether special techniques were needed.

We tested whether the 2 groups are comparable on baseline sociodemographic characteristics and baseline outcome measures using R package “CBCgrps” 2.8.2 [42]. The “twogrps” function examines discrepancies in categorical and continuous variables between two groups. It automatically assesses the distribution of the continuous variable and provides suitable descriptions accordingly. We included the variables as covariates in the models if significant differences were found. Between-group and group-by-time interaction effects were examined graphically and via linear mixed modeling for HbA1c and secondary outcomes. Changes in the outcomes over time in the wait-list control and FAMILY intervention groups were modeled and compared using piecewise linear mixed models, in which two time periods (0–3 and 3–6 months), group, and the group * time-period interactions were modeled as the fixed effects. When the difference in changing rates between two time periods was not significant within each group, linear mixed models were employed instead, in which time period (0–6 months), group, and the group * time-period interactions were modeled as the fixed effects. In all models, participant ID was treated as a random effect to take the within-subject correlations into account. All analyses were performed using R 4.2.1 and logistic LASSO regression was conducted using package “lme4” 1.1–33 [43].

## Results

### Demographic characteristics

A total of 23 dyads of participants were randomized and 11 dyads received the FAMILY intervention. As shown in Table 1, patients had a mean (SD) age of 56.2 (SD = 9.4) years and 52.2% were males. Most of them had high school educations or less (69.6%), and an annual household income under $25,000 (65.2%). Despite immigrating to the United States for 20.4 (SD = 11.9) years, 95.7% reported limited English proficiency. A significant difference in marital status was found between the two groups, with 54.5% of the FAMILY group and 100% of the control group being married (p = 0.014). The mean (SD) age of family and friends was 54.6 (11.2) years, with the control group significantly older than the FAMILY group (p = 0.003). About 52.2% of them were female and 73.9% were spouses of patients with diabetes. Similar to patients, most family and friends had high school educations or less (69.6%), with an annual household income under $25,000 (52.2%). On average, they had immigrated to the States for 15 (7.75, 30) years, and 95.7% reported limited English proficiency (Table 1). The complete deidentified data set for this study can be found in S5 File. Relevant data.

**Table 1:** Demographic characteristics by intervention condition at baseline.

Characteristics	Patients				Families and friends			
	Total (n = 23)	FAMILY (n = 11)	Control (n = 12)	Test^a^ p-value	Total (n = 23)	FAMILY (n = 11)	Control (n = 12)	Test^a^ p-value
Age in years, M (SD)	56.22 (9.44)	55.45 (11.45)	56.92 (7.62)	0.725	54.62 (11.18)	48.18 (11.04)	61.7 (5.98)	**0.003**
Gender, n (%)				1				0.525
Female	11 (47.83)	5 (45.45)	6 (50.00)		12 (52.17)	7 (63.60)	5 (41.70)	
Male	12 (52.17)	6 (54.50)	6 (50.00)		11 (47.83)	4 (36.40)	7 (58.30)	
Marital status, n (%)				**0.014**				1
Currently married or living as married	18 (78.26)	6 (54.50)	12 (100.00)		21 (91.30)	10 (90.90)	11 (91.70)	
Currently not married	5 (21.74)	5 (45.50)	0 (0.00)		2 (8.70)	1 (9.10)	1 (8.30)	
Education, n (%)				0.208				0.175
Never attended school or only attended kindergarten	1 (4.35)	0 (0.00)	1 (8.30)					
Elementary	10 (43.48)	4 (36.40)	6 (50.00)		7 (30.43)	3 (27.30)	4 (33.30)	
Some high school	2 (8.70)	0 (0.00)	2 (16.70)		1 (4.35)	0 (0.00)	1 (8.30)	
High school graduate	3 (13.04)	1 (9.10)	2 (16.70)		8 (34.78)	2 (18.20)	6 (50.00)	
Some college or technical school	4 (17.39)	3 (27.30)	1 (8.30)		3 (13.04)	2 (18.20)	1 (8.30)	
College graduate	3 (13.04)	3 (27.30)	0 (0.00)		3 (13.04)	3 (27.30)	0 (0.00)	
Refused to answer					1 (4.35)	1 (9.10)	0 (0.00)	
Annual household income, n (%)				0.348				0.077
<US $25,000	15 (65.22)	6 (54.50)	9 (75.00)		12 (52.17)	3 (27.30)	9 (75.00)	
US $25,000-US $55,000	5 (21.74)	2 (18.20)	3 (25.00)		7 (30.43)	4 (36.40)	3 (25.00)	
US $55,000	2 (8.70)	2 (18.20)	0 (0.00)		2 (8.70)	2 (18.20)	0 (0.00)	
Declined to answer or don’t know	1 (4.35)	1 (9.10)	0 (0.00)		2 (8.70)	2 (18.20)	0 (0.00)	
Employment status, n (%)				0.807				0.094
Employed full time	5 (21.74)	2 (18.20)	3 (25.00)		4 (17.39)	3 (27.30)	1 (8.30)	
Part-time (one job)	4 (17.39)	1 (9.10)	3 (25.00)		5 (21.74)	3 (27.30)	2 (16.70)	
Self-employed	1 (4.35)	1 (9.10)	0 (0.00)		2 (8.70)	1 (9.10)	1 (8.30)	
Not employed, not working	5 (21.74)	3 (27.30)	2 (16.70)		5 (21.74)	3 (27.30)	2 (16.70)	
Retired	8 (34.78)	4 (36.40)	4 (33.30)		6 (26.09)	0 (0.00)	6 (50.00)	
Foreign born, n (%)	23 (100.00)	11 (100.00)	12 (100.00)	1	23 (100.00)	11 (100.00)	12 (100.00)	1
English proficiency, n (%)				0.236				0.489
Very well	1 (4.35)	1 (9.10)	0 (0.00)		1 (4.35)	1 (9.10)	0 (0.00)	
Well	2 (8.70)	2 (18.20)	0 (0.00)		2 (8.70)	0 (0.00)	2 (16.70)	
Not well	12 (52.17)	4 (36.40)	8 (66.70)		13 (56.52)	7 (63.60)	6 (50.00)	
Not at all	8 (34.78)	4 (36.40)	4 (33.30)		6 (26.09)	2 (18.20)	4 (33.30)	
Duration of residency in years, M (SD)	20.37 (11.91)	18.00 (10.89)	22.54 (12.85)	0.370	15.00 (7.75, 30.00)	10.00 (7.00, 11.00)	22.50 (15.00, 30.00)	0.112
Relationship to patients, n (%)				-				0.105
Spouse	-	-	-	-	17 (73.91)	6 (54.50)	11 (91.70)	
Adult Children	-	-	-	-	3 (13.04)	3 (27.30)	0 (0.00)	
Others (siblings & aids)	-	-	-	-	3 (13.04)	2 (18.20)	1 (8.30)	
Hemoglobin a1c, Median (Q1, Q3)	7.70 (7.20, 8.65)	7.70 (7.35, 8.90)	7.75 (7.10, 8.53)	0.711	-	-	-	-
Weight (lbs.), M (SD) for patients/Median (Q1, Q3)for families and friends	158.65 (25.27)	157.27 (22.91)	159.92 (28.21)	0.807	144.00 (130.50, 159.00)	144.50 (130.00, 158.50)	144.00 (136.50, 162.00)	0.947

*Note*. normal data is summarized using M(SD), non-normal data is summarized using Median (Q1, Q3). ^a^Two sample t-test for continuous variables; Wilcoxon rank sum test for non-normal data; Fisher exact test for categorical variables. Significance is set at p < 0.05 and is in bold.

### Feasibility and acceptability outcomes

#### Feasibility

**Fig 1:**
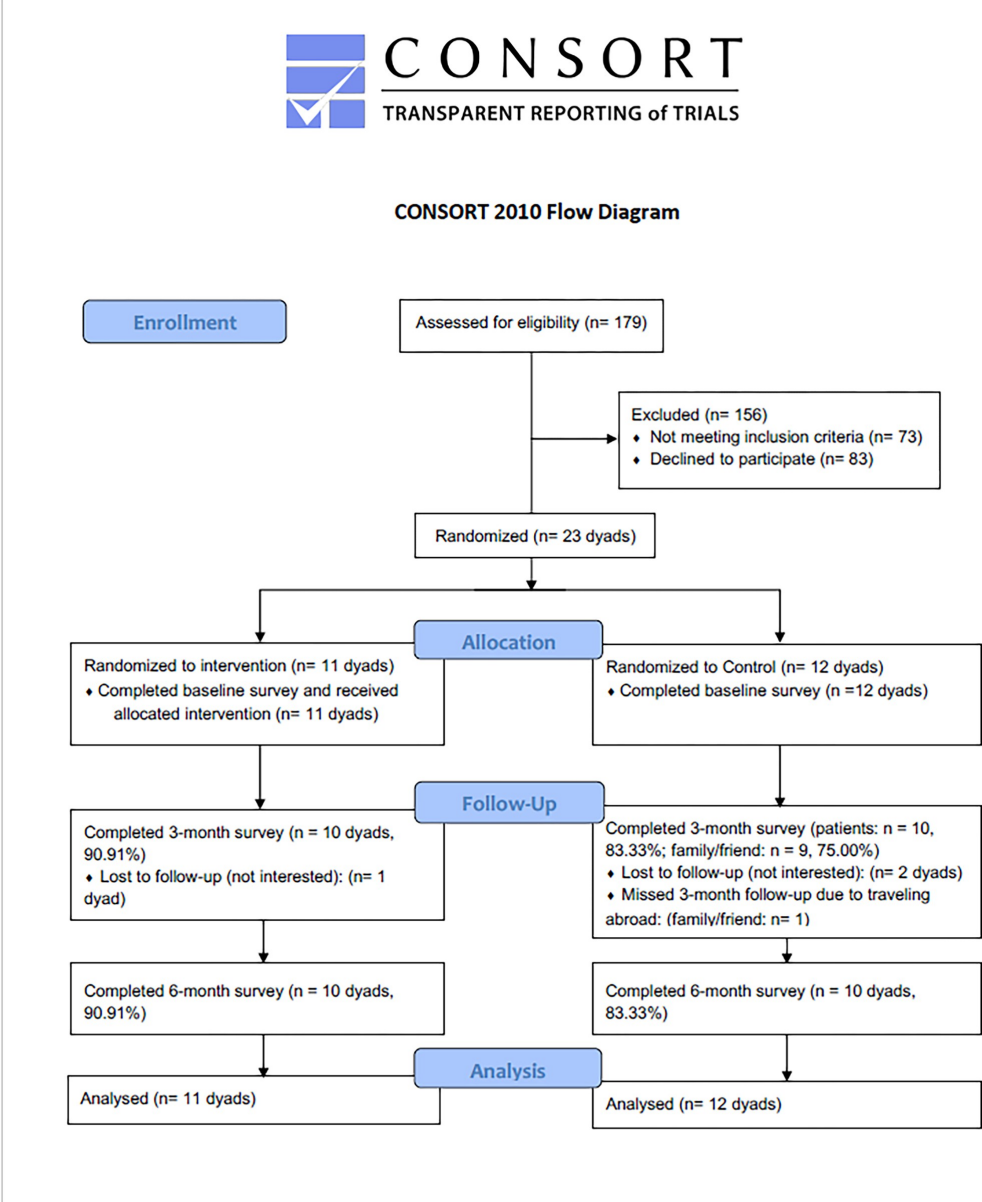
Participant enrollment flow.

The feasibility was measured by recruitment status, retention rates, and video watch rates. Fig 1 shows the flow of participants through the trial. From April 2021 to July 2021, we called 179 potential patients for screening and enrolled 23 dyads of participants in this study. The reasons for not participating are being ineligible and declining to participate. Regarding the retention rates at the 3-month and 6-month follow-ups, there was no statistically significant difference between the intervention group and the control group for both the patients (90.91% vs 83.3%, p = 0.589); and their families/friends (3-month: 90.9% vs 75%, p = 0.313; 6-month: 90.9% vs 83.3%, p = 0.589). The mean (SD) video watch rate was 76.8% (7%). The video watch rate over the 12-week intervention ranged from 65% to 90%, with diet-related videos ("Diabetes Diet 101", "Grocery Shopping at Chinese Supermarkets" and "Healthy Eating during Chinese holidays") being the most watched by both patients and families/friends.

#### Acceptability

Out of a possible score of 10 with higher scores reflecting greater satisfaction, the mean (SD) overall satisfaction with the intervention of patients and families/friends was 9.4 (1.0) and 10 (0.0), respectively. Table 2 shows participants’ feedback on each satisfaction item. Most participants agreed or strongly agreed on the ease of receiving and viewing diabetes videos, the videos provided helpful information about healthy diet and physical activity, and enhanced their loved ones’ or their own confidence in T2D management. They also strongly agreed or agreed that they would be willing to receive similar interventions in the future, recommend this intervention to others with diabetes, and prefer video-based diabetes education to in-person education in their doctor’s office.

**Table 2:** Satisfaction survey results.

	Patients (n = 10)					Families/Friends (n = 10)				
To what extent do you agree with the following statements?	Strongly agree, n (%)	Agree, n (%)	Neutral, n (%)	Disagree or strongly disagree, n (%)	Not applicable, n (%)	Strongly agree, n (%)	Agree, n (%)	Neutral, n (%)	Disagree or strongly disagree, n (%)	Not applicable, n (%)
It was easy to receive and view the WeChat diabetes videos from the research team	7 (70)	3 (30)	0 (0)	0 (0)	0 (0)	5 (50)	5 (50)	0 (0)	0 (0)	0 (0)
I found this program to be helpful for providing me more information about healthy diet	7 (70)	3 (30)	0 (0)	0 (0)	0 (0)	5 (50)	5 (50)	0 (0)	0 (0)	0 (0)
I found this program to be helpful for providing me more information about physical activity	7 (70)	3 (30)	0 (0)	0 (0)	0 (0)	5 (50)	5 (50)	0 (0)	0 (0)	0 (0)
I found this program to be helpful at motivating me to take my diabetes medication as prescribed	7 (70)	3 (30)	0 (0)	0 (0)	0 (0)	5 (50)	4 (40)	1 (10)	0 (0)	0 (0)
I found this program to be helpful at motivating me to check my blood sugar as recommended	7 (70)	3 (30)	0 (0)	0 (0)	0 (0)	5 (50)	4 (40)	1 (10)	0 (0)	0 (0)
I found this program to be helpful at increasing my confidence to manage my diabetes	7 (70)	3 (30)	0 (0)	0 (0)	0 (0)	5 (50)	5 (50)	0 (0)	0 (0)	0 (0)
I would be willing to join similar programs in the future to help me (my love ones) manage my (their) diabetes	7 (70)	3 (30)	0 (0)	0 (0)	0 (0)	5 (50)	5 (50)	0 (0)	0 (0)	0 (0)
I would recommend this program to my friends/family that have diabetes	7 (70)	3 (30)	0 (0)	0 (0)	0 (0)	5 (50)	5 (50)	0 (0)	0 (0)	0 (0)
I prefer to receive diabetes education via WeChat than scheduling appointment and going to doctor’s office	7 (70)	3 (30)	0 (0)	0 (0)	0 (0)	5 (50)	5 (50)	0 (0)	0 (0)	0 (0)

### Linear mixed model for secondary outcomes

#### Patient outcomes

Table 3 shows the changes in patients’ secondary outcomes over time. From baseline to 6 months, HbA1c decreased by 1.23% (95% CI [-2.21%, -0.24%], p = 0.014) for the FAMILY intervention group and by 0.88% (95% CI [-1.81%, 0.05%], p = 0.064) for the control group, with no statistically significant difference between groups (difference of change = -0.35%, 95% CI [-1.70%, 1.00%], p = 0.611). That is, HbA1c did not decrease as a result of the FAMILY intervention. There was a trend for a decline in patients’ body weight at 6 months in both the FAMILY intervention group (change = -11.7lb, 95% CI [-24.7, 1.4], p = 0.079) and the control group (change = -4.8lb, 95% CI [-17.7, 8.2], p = 0.472), with a 6.9lbs between-group difference (95% CI [-25.28, 11.43], p = 0.459). Similarly, none of the changes in other secondary outcomes, such as dietary intake, physical activity, self-efficacy, self-management, support, and diabetes stress, differed significantly between groups. With regard to within group difference at 6 months, the FAMILY intervention patients had a significant decrease in HbA1c (change = -1.23%, 95% CI [-2.21%, -0.24%], p = 0.014) and sugary drink intake (change = -0.31, 95% CI [-0.58, -0.04], p = 0.024), and a significant increase in self-management (change = 10.5, 95% CI [2.56, 17.53], p = 0.009) and emotional support (change = 0.63, 95% CI [0.07, 1.19], p = 0.028).

**Table 3:** Results of linear mixed regression analyses: Total change from baseline to 3 months, 3 months to 6 months, and baseline to 6 months in patients’ primary and secondary outcomes.

	FAMILY			Control			Difference of change (FAMILY—Control)		
	Estimate	95%CI	p-value	Estimate	95%CI	p-value	Estimate	95%CI	p-value
Hemoglobin A1c									
0 to 3 Months	-0.61	(-1.11, -0.12)	**0.014**	-0.44	(-0.90, 0.03)	0.064	-0.18	(-0.85, 0.50)	0.611
3 to 6 Months	-0.61	(-1.11, -0.12)	**0.014**	-0.44	(-0.90, 0.03)	0.064	-0.18	(-0.85, 0.50)	0.611
0 to 6 Months	-1.23	(-2.21, -0.24)	**0.014**	-0.88	(-1.81, 0.05)	0.064	-0.35	(-1.70, 1.00)	0.611
Weight (lbs.)									
0 to 3 Months	-5.84	(-12.35, 0.67)	0.079	-2.37	(-8.84, 4.09)	0.472	-3.46	(-12.64, 5.71)	0.459
3 to 6 Months	-5.84	(-12.35, 0.67)	0.079	-2.37	(-8.84, 4.09)	0.472	-3.46	(-12.64, 5.71)	0.459
0 to 6 Months	-11.68	(-24.71, 1.35)	0.079	-4.75	(-17.68, 8.18)	0.472	-6.93	(-25.28, 11.43)	0.459
Fruits intake									
0 to 3 Months	0.04	(-0.18, 0.26)	0.716	0.04	(-0.18, 0.25)	0.752	0.01	(-0.30, 0.32)	0.970
3 to 6 Months	0.04	(-0.18, 0.26)	0.716	0.04	(-0.18, 0.25)	0.752	0.01	(-0.30, 0.32)	0.970
0 to 6 Months	0.08	(-0.36, 0.52)	0.716	0.07	(-0.37, 0.51)	0.752	0.01	(-0.61, 0.63)	0.970
Vegetable intake									
0 to 3 Months	0.20	(-0.09, 0.49)	0.170	0.29	(0.00, 0.57)	0.051	-0.08	(-0.49, 0.33)	0.691
3 to 6 Months	0.20	(-0.09, 0.49)	0.170	0.29	(0.00, 0.57)	0.051	-0.08	(-0.49, 0.33)	0.691
0 to 6 Months	0.41	(-0.17, 0.99)	0.170	0.57	(0.00, 1.15)	0.051	-0.17	(-0.98, 0.65)	0.691
Refined grains intake									
0 to 3 Months	-0.05	(-0.24, 0.15)	0.633	-0.16	(-0.35, 0.04)	0.111	0.11	(-0.16, 0.38)	0.433
3 to 6 Months	-0.05	(-0.24, 0.15)	0.633	-0.16	(-0.35, 0.04)	0.111	0.11	(-0.16, 0.38)	0.433
0 to 6 Months	-0.09	(-0.48, 0.29)	0.633	-0.31	(-0.70, 0.07)	0.111	0.22	(-0.33, 0.76)	0.433
Whole wheat intake									
0 to 3 Months	0.08	(-0.22, 0.38)	0.592	-0.16	(-0.46, 0.14)	0.291	0.24	(-0.18, 0.66)	0.261
3 to 6 Months	0.08	(-0.22, 0.38)	0.592	-0.16	(-0.46, 0.14)	0.291	0.24	(-0.18, 0.66)	0.261
0 to 6 Months	0.16	(-0.44, 0.77)	0.592	-0.32	(-0.91, 0.27)	0.291	0.48	(-0.36, 1.33)	0.261
Sugary drinks intake_									
0 to 3 Months	-0.15	(-0.29, -0.02)	**0.024**	-0.11	(-0.24, 0.02)	0.096	-0.04	(-0.23, 0.14)	0.637
3 to 6 Months	-0.15	(-0.29, -0.02)	**0.024**	-0.11	(-0.24, 0.02)	0.096	-0.04	(-0.23, 0.14)	0.637
0 to 6 Months	-0.31	(-0.58, -0.04)	**0.024**	-0.22	(-0.48, 0.04)	0.096	-0.09	(-0.46, 0.28)	0.637
Starchy intake									
0 to 3 Months	-0.04	(-0.14, 0.07)	0.513	0.00	(-0.10, 0.11)	0.970	-0.04	(-0.19, 0.11)	0.621
3 to 6 Months	-0.04	(-0.14, 0.07)	0.513	0.00	(-0.10, 0.11)	0.970	-0.04	(-0.19, 0.11)	0.621
0 to 6 Months	-0.07	(-0.28, 0.14)	0.513	0.00	(-0.20, 0.21)	0.970	-0.07	(-0.37, 0.22)	0.621

Physical activity									
0 to 3 Months	538.35	(-670.10, 1746.80)	0.383	828.19	(-367.85, 2024.22)	0.175	-289.84	(-1990.08, 1410.41)	0.738
3 to 6 Months	538.35	(-670.10, 1746.80)	0.383	828.19	(-367.85, 2024.22)	0.175	-289.84	(-1990.08, 1410.41)	0.738
0 to 6 Months	1076.70	(-1340.20, 3493.59)	0.383	1656.37	(-735.69, 4048.44)	0.175	-579.67	(-3980.17, 2820.82)	0.738
Self-efficacy									
0 to 3 Months	0.19	(-0.34, 0.72)	0.481	0.21	(-0.30, 0.73)	0.420	-0.02	(-0.76, 0.72)	0.952
3 to 6 Months	0.19	(-0.34, 0.72)	0.481	0.21	(-0.30, 0.73)	0.420	-0.02	(-0.76, 0.72)	0.952
0 to 6 Months	0.38	(-0.68, 1.44)	0.481	0.43	(-0.61, 1.46)	0.420	-0.05	(-1.52, 1.43)	0.952
Self-management									
0 to 3 Months	5.02	(1.28, 8.77)	**0.009**	3.58	(-0.13, 7.29)	0.059	1.45	(-3.82, 6.72)	0.590
3 to 6 Months	5.02	(1.28, 8.77)	**0.009**	3.58	(-0.13, 7.29)	0.059	1.45	(-3.82, 6.72)	0.590
0 to 6 Months	10.05	(2.56, 17.53)	**0.009**	7.15	(-0.26, 14.57)	0.059	2.89	(-7.64, 13.43)	0.590
Diabetes-specific support*									
0 to 3 Months	2.19	(-3.31, 7.68)	0.436	8.27	(2.88, 13.67)	**0.003**	-6.09	(-13.79, 1.62)	0.121
3 to 6 Months	0.96	(-4.60, 6.52)	0.735	-1.35	(-6.87, 4.16)	0.631	2.31	(-5.52, 10.14)	0.563
0 to 6 Months	3.15	(-4.70, 11.00)	0.432	6.92	(-0.73, 14.58)	0.076	-3.78	(-14.74, 7.19)	0.499
Emotional support									
0 to 3 Months	0.31	(0.03, 0.59)	**0.028**	0.32	(0.04, 0.60)	**0.025**	0.00	(-0.40, 0.39)	0.982
3 to 6 Months	0.31	(0.03, 0.59)	**0.028**	0.32	(0.04, 0.60)	**0.025**	0.00	(-0.40, 0.39)	0.982
0 to 6 Months	0.63	(0.07, 1.19)	**0.028**	0.64	(0.08, 1.19)	**0.025**	-0.01	(-0.80, 0.78)	0.982
Diabetes stress									
0 to 3 Months	-0.25	(-0.60, 0.10)	0.156	-0.34	(-0.68, 0.00)	0.052	0.08	(-0.40, 0.57)	0.733
3 to 6 Months	-0.25	(-0.60, 0.10)	0.156	-0.34	(-0.68, 0.00)	0.052	0.08	(-0.40, 0.57)	0.733
0 to 6 Months	-0.50	(-1.20, 0.19)	0.156	-0.67	(-1.35, 0.01)	0.052	0.17	(-0.80, 1.14)	0.733

Note. *Results of piecewise linear mixed model. All models were adjusted for marital status at baseline. Significance set at *p* < 0.05 and is in bold. About 45% of patients in the FAMILY intervention group and 50% in the control group had complete secondary outcome data.

#### Families/Friends outcomes

Table 4 shows the changes in family and friends’ body weight, dietary intake, physical activity, social support, and diabetes knowledge over time. The changes in body weight, physical activity, social support, and diabetes knowledge between the two groups did not differ significantly from baseline to 6 months period. The FAMILY intervention group experienced greater reductions in dietary intake across all food types compared to the control group. Specifically, significant reductions were observed in fruits, vegetables, and whole wheat from 0 to 6 months, with a difference of change of -1.21 (95% CI [-2.01, -0.41], p = 0.003), -1.36 (95% CI [-2.52, -0.19], p = 0.023), and -0.53 (95% CI [-0.92, -0.14], p = 0.008), respectively. Additionally, the FAMILY group had significant reductions in starch intake from 3 to 6 months, with a difference of change of -0.18 (95% CI [-0.35, 0.00], p = 0.045), compared to the control group. As for changes within the FAMILY intervention group over the 6 months, families/friends had a significant decrease in refined grains intake (change = -0.68, 95% CI [-1.18, -0.17], p = 0.009), and an increase in emotional support (change = 0.70, 95% CI [0.06, 1.34], p = 0.033) and diabetes knowledge (change = 0.24, 95% CI [0.11, 0.37], p < 0.001).

**Table 4:** Results of linear mixed regression analyses: Total change from baseline to 3 months, 3 months to 6 months, and baseline to 6 months in family and friends’ outcomes.

	FAMILY			Control			Difference of change (FAMILY—Control)		
	Estimate	95%CI	p-value	Estimate	95%CI	p-value	Estimate	95%CI	p-value
Weight (lbs.)									
0 to 3 Months	-1.29	(-12.62, 10.03)	0.823	-2.44	(-14.53, 9.65)	0.692	1.15	(-15.42, 17.71)	0.892
3 to 6 Months	-1.29	(-12.62, 10.03)	0.823	-2.44	(-14.53, 9.65)	0.692	1.15	(-15.42, 17.71)	0.892
0 to 6 Months	-2.59	(-25.24, 20.06)	0.823	-4.88	(-29.06, 19.30)	0.692	2.29	(-30.84, 35.43)	0.892
Fruits intake									
0 to 3 Months	-0.09	(-0.36, 0.18)	0.515	0.51	(0.22, 0.81)	**0.001**	-0.60	(-1.00, -0.20)	**0.003**
3 to 6 Months	-0.09	(-0.36, 0.18)	0.515	0.51	(0.22, 0.81)	**0.001**	-0.60	(-1.00, -0.20)	**0.003**
0 to 6 Months	-0.18	(-0.71, 0.36)	0.515	1.03	(0.43, 1.62)	**0.001**	-1.21	(-2.01, -0.41)	**0.003**
Vegetable intake									
0 to 3 Months	-0.09	(-0.48, 0.30)	0.661	0.59	(0.16, 1.02)	**0.008**	-0.68	(-1.26, -0.09)	**0.023**
3 to 6 Months	-0.09	(-0.48, 0.30)	0.661	0.59	(0.16, 1.02)	**0.008**	-0.68	(-1.26, -0.09)	**0.023**
0 to 6 Months	-0.18	(-0.96, 0.61)	0.661	1.18	(0.31, 2.05)	**0.008**	-1.36	(-2.52, -0.19)	**0.023**
Refined grains intake									
0 to 3 Months	-0.34	(-0.59, -0.09)	**0.009**	-0.20	(-0.47, 0.08)	0.169	-0.14	(-0.52, 0.23)	0.455
3 to 6 Months	-0.34	(-0.59, -0.09)	**0.009**	-0.20	(-0.47, 0.08)	0.169	-0.14	(-0.52, 0.23)	0.455
0 to 6 Months	-0.68	(-1.18, -0.17)	**0.009**	-0.39	(-0.95, 0.17)	0.169	-0.29	(-1.04, 0.46)	0.455
Whole wheat intake*									
0 to 3 Months	-0.02	(-0.28, 0.24)	0.888	0.58	(0.27, 0.88)	**0.000**	-0.59	(-0.10, -0.19)	**0.004**
3 to 6 Months	-0.08	(-0.34, 0.19)	0.578	-0.14	(-0.45, 0.17)	0.386	0.06	(-0.35, 0.47)	0.765
0 to 6 Months	-0.09	(-0.36, 0.17)	0.484	0.44	(0.15, 0.73)	**0.003**	-0.53	(-0.92, -0.14)	**0.008**
Sugary drinks intake									
0 to 3 Months	-0.11	(-0.27, 0.04)	0.148	-0.09	(-0.26, 0.07)	0.270	-0.02	(-0.25, 0.21)	0.861
3 to 6 Months	-0.11	(-0.27, 0.04)	0.148	-0.09	(-0.26, 0.07)	0.270	-0.02	(-0.25, 0.21)	0.861
0 to 6 Months	-0.23	(-0.53, 0.08)	0.148	-0.19	(-0.52, 0.14)	0.270	-0.04	(-0.49, 0.41)	0.861
Starch intake*									
0 to 3 Months	-0.02	(-0.14, 0.09)	0.722	-0.15	(-0.28, -0.02)	**0.020**	0.13	(-0.04, 0.31)	0.131
3 to 6 Months	-0.05	(-0.17, 0.06)	0.375	0.13	(-0.01, 0.26)	0.059	-0.18	(-0.35, 0.00)	**0.045**
0 to 6 Months	-0.07	(-0.24, 0.09)	0.393	-0.03	(-0.21, 0.16)	0.772	-0.05	(-0.29, 0.20)	0.717
Physical activity									
0 to 3 Months	382.92	(-209.05, 974.89)	0.205	164.86	(-493.12, 822.83)	0.623	218.07	(-666.95, 1103.08)	0.629
3 to 6 Months	382.92	(-209.05, 974.89)	0.205	164.86	(-493.12, 822.83)	0.623	218.07	(-519.36, 2442.24)	0.629
0 to 6 Months	765.84	(-418.10, 1949.79)	0.205	329.71	(-986.25, 1645.67)	0.623	436.13	(-1333.90, 2206.16)	0.629

Diabetes-specific support									
0 to 3 Months	4.56	(-0.06, 9.17)	0.053	4.61	(-0.36, 9.58)	0.069	-0.05	(-6.83, 6.73)	0.988
3 to 6 Months	4.56	(-0.06, 9.17)	0.053	4.61	(-0.36, 9.58)	0.069	-0.05	(-6.83, 6.73)	0.988
0 to 6 Months	9.12	(-0.12, 18.35)	0.053	9.22	(-0.72, 19.16)	0.069	-0.10	(-13.67, 13.47)	0.988
Emotional support									
0 to 3 Months	0.35	(0.03, 0.67)	**0.033**	0.60	(0.25, 0.95)	**0.001**	-0.25	(-0.73, 0.22)	0.300
3 to 6 Months	0.35	(0.03, 0.67)	**0.033**	0.60	(0.25, 0.95)	**0.001**	-0.25	(-0.73, 0.22)	0.300
0 to 6 Months	0.70	(0.06, 1.34)	**0.033**	1.20	(0.50, 1.91)	**0.001**	-0.50	(-1.46, 0.45)	0.300
Diabetes knowledge									
0 to 3 Months	0.12	(0.05, 0.19)	**0.000**	0.07	(0.00, 0.14)	**0.044**	0.05	(-0.05, 0.14)	0.330
3 to 6 Months	0.12	(0.05, 0.19)	**0.000**	0.07	(0.00, 0.14)	**0.044**	0.05	(-0.05, 0.14)	0.330
0 to 6 Months	0.24	(0.11, 0.37)	**0.000**	0.14	(0.00, 0.29)	**0.044**	0.10	(-0.10, 0.29)	0.330

Note. *Results of piecewise linear mixed model. All models were adjusted for age at baseline; models testing changes in vegetable intake and physical activity were adjusted for their baseline measures. Significance is set at *p* < 0.05 and is in bold. About 82% of families/friends in the FAMILY intervention group and 75% in the control group had complete secondary outcome data.

## Discussion

Study findings indicated that a culturally-tailored, family-based mHealth intervention is feasible and acceptable among low-income Chinese immigrant families with T2D in New York City. The retention rates were above 80% for both patient and families/friend participants follow-ups at 6 months. On average, participants watched 76.7% (SD = 7.0%) of the videos. T2D patients and families/friends rated satisfaction as 9.4 and 10 out of 10. Despite no between-group differences, the intervention group had significantly lower HbA1c (p = 0.014) and improved self-management (p = 0.009), and lost almost 12 lbs. on average at 6 months (p = 0.079).

Participants actively engaged with the diabetes self-management education (DSME) videos with a mean watch rate of 76.7%, exceeding the mean articles read rate (38.3%) in a similar pilot study using WeChat [44]. The higher engagement observed in this study may be attributed to the videos’ convenience and brevity. Video telecare education is found to be as effective as in-person education, can be easily adapted to patients’ cultural backgrounds, can be repeated and viewed at patients’ convenience, and can be easily shared with patients’ support systems [45]. Similarly, we found a high video watch rate (92%) in our other study on a patient-based diabetes management intervention delivered by WeChat videos (hereafter CARE), supporting the feasibility of this intervention delivery method [33]. The slightly higher watch rates in the CARE study may relate to participants’ rapport with the study team. Many of the participants in the CARE study have participated in prior studies led by our study team and they have established trust. Additionally, the CARE videos were sent at the beginning of the pandemic in NYC (March 2020), when most of these participants were furloughed or lost their jobs due to COVID-19. Also, the state-wide stay-at-home orders during the beginning of the pandemic and people being anxiously on their phones most of the time for more up-to-date information may contribute to a higher video watch rate in the CARE study. Increased social media and technology utilization during the pandemic was evident both in the United States and globally [46,47]. This study was conducted completely remotely over phone calls from June to December 2021 when people were returning to their regular routines.

Additionally, the current study found a preference for video-based diabetes education over in-person programs and higher satisfaction (9.4 and 10 out of 10) than another in-person DSME programs, where 73.9% of the participants were satisfied with the in-person diabetes sessions [48]. It is worth noting that families/friends were highly satisfied with our mHealth intervention, whereas low family participation rates were identified as the main obstacle to family-based in-person diabetes education [49]. The preference for video-based interventions over in-person programs may stem from the convenience they offer, as they eliminate the need for travel. Additionally, participants can view the videos at their own pace and rewatch them as needed to revisit specific information. A prior study showed that illness, work commitments, childcare, weather conditions, holidays, and forgetfulness were the common barriers for patients with T2D to attend in-person diabetes programs [50]. Short educational videos delivered via social media can address these barriers and reach out to those without access to in-person DSME programs.

We observed some interesting variations in the watching rate of videos on different topics, with diet-related education drawing the greatest interest from participants. "Diabetes Diet 101", "Grocery Shopping at Chinese Supermarkets", and "Healthy Eating during Chinese holidays" were the most watched videos. Participants were less interested in videos related to mental health such as "Stress Management" and "Emotional Eating". The possible reason is that mental health is still a stigmatized and rarely discussed topic in Chinese culture and thus people lack mental health knowledge [51]. Thus, some participants are hesitant to admit they suffer from anxiety or emotional eating.

Given the relatively small sample size and pilot nature of this study, we did not observe significant group difference in changes in primary or secondary outcomes. Although the HbA1c of the FAMILY group decreased significantly over the 6 months, there was no statistically significant difference between the intervention and control groups. The intervention group also showed significant changes, with non-significant between-group differences, in sugary drink intake, self-management, and emotional support for patients, as well as refined grain intake, social support, and diabetes knowledge for families/friends. The non-significant findings could be due to the small sample size of this pilot study, which is anticipated as our primary goal is to examine the feasibility and acceptability of the FAMILY intervention. Another explanation could be that the study was conducted during the COVID-19 pandemic, which may have influenced people’s health status and lifestyles. COVID-19 poses a higher risk of causing more serious health complications for people with diabetes [52]. Besides this direct effect, they also faced challenges undertaking outdoor activities and securing sufficient food during the pandemic. For example, people may consider their neighborhoods unsafe due to anti-Asian hate crimes [53], so they stop exercising outside. Food security and access to nutritious foods have also been negatively affected by COVID-19 [54]. Food insecurity was rated as the top concern by Asian New Yorkers during the pandemic due to limited supplies, closures of grocery stores, price hikes, and unemployment [55]. Further, households with chronic conditions were more likely to report rising food prices and use emergency food services than households without chronic conditions [56,57]. Despite the non-significant results, we found a difference in weight loss of 6.9 lbs. between the groups with large standard deviation. In light of this result, a mobile-based health education program that involves family members may likely improve health outcomes, but further research with larger sample sizes is needed to examine the efficacy of such mobile-based family interventions.

Our study is unique in that it involves family and friends in diabetes self-management education. In East Asian culture, people tend to share different dishes and have communal eating styles. Women are usually responsible for preparing food and caring for their families [58]. It is therefore critical to educate family members responsible for cooking about meal plans for diabetes and healthy food choices instead of only educating the patient. In addition, dining out with others is a common way to build interpersonal relationships. East Asians with T2D reported feeling ashamed if they need to follow a diabetes diet in the presence of others since this may elicit special attention [59]. Thus, it is crucial to keep family and friends informed about diabetes care to foster a supportive environment for people with diabetes.

The preliminary data from this study also provides several critical implications for future large-scale trial planning. Using the observed reductions in FAMILY (-1.23%) and control (-0.88%) groups and SD = 0.49% of HbA1c at 6 months in our preliminary study, based on a two-sample two-sided t-test, n = 32 (per group) will be required to detect a minimum group difference of 0.35% in HbA1c with a power of 80% and a type I error of 0.05. We achieved a 91% retention rate in the FAMILY intervention group and 83% in the control group in this pilot study. With a conservative retention rate of 80%, we will need to recruit 80 (40/group) participants to yield a final sample of at least 64 (32/group). In addition, this study provided important lessons with regard to involving family members and dyads in clinical trials. Recruiting participants in clinical trials has always been one of the most challenging parts. Yet, recruiting dyads poses additional challenges. In this pilot study, we had some patient participants who were interested in joining the study but they could not identify a family member/friend who was willing to join as a dyad. Also, in this study, we first identified patient participants and screened them for eligibility. Then we waited for them to contact their family member/friend to see whether they would be interested in joining and if so, they will call the research team to confirm and provide contact information for the family/friend participants. This process may take a few weeks before we can officially enroll this dyad. Thus, in future studies, researchers need to plan extra time and increase the patient pool to account for these potential challenges.

### Strength and limitations

The study has a few limitations. First, the sample size is small, which makes it challenging to detect statistically significant differences between the control group and the intervention group. Also, because the watch rate data was collected in an aggregated manner, it was impossible to ascertain whether responses came from patients or their family/friend groups or to identify the individual viewers of the videos. We could not examine whether patients had a higher watching rate than families/friends participants. While self-report satisfaction rates indicate low participant burdens, they may be influenced by social desirability bias. Therefore, a larger sample size and measures to differentiate the video-viewing behavior of participants may be helpful in future studies. Another study limitation is the notable age disparity between families/friends in the control group and the intervention group. Although we considered age in our analysis, these differences could still potentially influence our findings. Notably, the control group consisted mainly of patients’ spouses who may spend more time together and receive greater support, which could explain their better outcomes. The relatively younger age of the intervention group might have impacted the effectiveness of our intervention. To address this, future studies could benefit from a larger sample size and stratified randomization to enhance the balance between the intervention and control groups.

There are also several strengths to highlight. Our study is among the first studies to engage family members in diabetes intervention in Chinese Americans with T2D. It may serve as a model for other chronic conditions and racial and ethnic minorities. Our study focused on a significantly understudied immigrant group, the majority of whom had low socioeconomic status and limited education and English proficiency. While most of the existing mHealth studies excluded these underserved communities, our study demonstrated that it was feasible to engage these communities in a mHealth intervention. Although there is widespread acknowledgment of diabetes disparities within minoritized populations, there are significant knowledge gaps in understanding how to culturally adapt and implement evidence-based interventions to address the needs of these diverse underserved groups. Our pilot study suggests that we could potentially leverage family/friends and basic text messaging technology to help disseminate evidence-based interventions and support minoritized patients in diabetes management.

## Conclusions

The present study adds to the expanding literature on family-based diabetes self-management education by establishing the feasibility and acceptability of a family-oriented mHealth intervention for Chinese Americans with T2D. This intervention was culturally tailored to meet the needs of this historically underserved and understudied population. Our results demonstrate that this family-based mHealth intervention is feasible and acceptable among low-income, limited English-proficient Chinese families with type 2 diabetes in New York City. Significant changes in HbA1c and self-management in the intervention group indicate this intervention may have potential efficacy. The small sample size and lack of statistical power of this study warrant future studies with a larger sample size to test its efficacy.

## Supporting information

S1 File CONSORT checklist. (DOC)

S2 File Clinical study checklist. (DOCX)

S3 File Study protocol. (DOCX)

S4 File IRB approval. (PDF)

S5 File Relevant data. (XLSX)
