# Supplementary material for: Feasibility of a family-oriented mHealth intervention for Chinese Americans with type 2 diabetes: A pilot randomized control trial
Source: PLoS One. 2024 Mar 11;19(3):e0299799. doi: 10.1371/journal.pone.0299799 (PMC10927140; doi:10.1371/journal.pone.0299799)
Supplement: S3 File — (DOCX) [file pone.0299799.s003.docx]

**Feasibility of a Family-oriented mHealth Intervention for Chinese Americans with Type 2 Diabetes**

**Principal Investigator:** Lu Hu, PhD

NYU School of Medicine

Department of Population Health

180 Madison Ave

New York, NY 10016

646-501-3438

[Hu.Lu@nyulangone.org](mailto:Hu.Lu@nyulangone.org)

**Protocol Number:** s19-01275

**Version #:** Version 3.26.2021

Table of Contents

[1. Abstract 3](#_Toc59205001)

[2. Purpose of the Study and Background 3](#_Toc59205002)

[2.1 Background 3](#_Toc59205003)

[2.2 Aims 4](#_Toc59205004)

[3. METHODS and procedures 5](#_Toc59205005)

[3.1 Study Design 5](#_Toc59205006)

[3.2 Sites 5](#_Toc59205007)

[3.3. Characteristics of the Research Population 5](#_Toc59205008)

[4. Procedures 6](#_Toc59205009)

[4.1 Patient Recruitment 6](#_Toc59205010)

[4.2 Patient Consent 6](#_Toc59205011)

[4.3 Study Procedures 6](#_Toc59205012)

[5. Risk/Benefit Assessment 9](#_Toc59205013)

[5.1 Potential Risks 9](#_Toc59205014)

[5.2 Benefits 9](#_Toc59205015)

[6. Confidentiality 9](#_Toc59205016)

[7. Data Analysis and Data Monitoring 10](#_Toc59205017)

[7.1 Data Analysis 10](#_Toc59205018)

[7.2 Data Monitoring 11](#_Toc59205019)

[8. Investigators Qualifications 12](#_Toc59205020)

[9. Study Records Retention and Data Sharing 12](#_Toc59205021)

[10. References 14](#_Toc59205022)

# 1. Abstract

Chinese Americans are one of the fastest growing immigrant groups in the US, who suffer disproportionately high type 2 diabetes (T2D) burden and have poorly controlled T2D. Given the high economic and societal burden of T2D and rapid population growth in Chinese Americans, there is an urgent need for research to develop effective interventions to address T2D burden in this population. Recent evidence suggests the importance of involving and empowering family members in diabetes intervention and calls for family intervention. A mobile health approach such as short message service (SMS) might be a promising way to deliver such family-oriented interventions to the target population given prior studies suggests that Chinese Americans often report challenges to attend in-person lifestyle counseling because of long working hours and lack of sick time from work. A SMS-based intervention provides the flexibility of allowing them to view the intervention at a time and place convenient to them. However, such intervention has not been developed for or tested in Chinese Americans with T2D. This study will serve as the first step to explore an alternative approach for managing T2D in this group. The goal of this pilot study is to examine the feasibility and acceptability of a family-oriented SMS intervention in 30 pairs of Chinese Americans with T2D and their family members. Participants will be randomized to one of 2 arms (n=15 pairs each): 1) wait-list control and 2) SMS intervention. Both groups will continue to receive standard of care treatment for their T2D. The SMS group will receive brief lifestyle counseling videos via SMS links. At the end of the study, the wait-list control group will be provided the opportunity to receive the counseling videos. Measurements will be obtained at baseline, 3, and 6 months. The primary outcome is feasibility and acceptability, and secondary outcomes include HbA1c, self-efficacy, and diabetes self-management behaviors. Findings from this pilot study will inform a larger full-scale R01 efficacy trial of the SMS intervention, and ultimately, an implementation study in the real-world setting. This project can serve as a program model for other chronic disease interventions in Chinese Americans that require lifestyle modification (e.g., prediabetes, hypertension), or for disparities research in other high-risk immigrant populations (e.g., South Asians, Hispanic Americans).

# 2. Purpose of the Study and Background

## 2.1 Background

Chinese Americans are one of the fastest growing immigrant groups in the United States.^1–3^ Multiple studies show that, compared to non-Hispanic whites, Chinese Americans suffer disproportionately higher rates of type 2 diabetes (T2D),^4–8^ have poorer self-management and glycemic control,^9–13^ and are at elevated risk of developing renal complications.^14^ Lack of culturally and linguistically sensitive healthcare providers and programs are the most commonly cited reasons for disparities in T2D care and health outcomes in Chinese Americans.^15–17^ High rates of limited English proficiency, poor access to healthcare and patient-provider communication barriers have played a critical role in prevention and management of T2D for this population.^15–17^ Given the substantial and growing limited English proficient Chinese American population in the United States and the continued rising prevalence of T2D, there is an urgent need to develop, culturally-adapt, and test evidence-based interventions for diabetes control in this population.^18^

While the majority of current diabetes interventions only involved patients, there is a growing body of evidence demonstrating the importance of educating and empowering family members in this process.^19–21^ The success of diabetes management depends largely on the social and environment contexts in which patients live and perform diabetes self-care.^20,22^ When patients with T2D live in a supportive family context (e.g., the family embraces a lifestyle consistent with the needs of the patient), they are more likely to receive social and emotional support for self-management, adhere to diabetes self-care requirements, and achieve better glycemic control.^23^ Patients with T2D living in non-supportive family environments (e.g., family members undermine the patient’s efforts, use negative communication strategies) have more difficulty initiating and sustaining recommended diabetes self-care behaviors, experience more diabetes-related distress, and report lower self-efficacy for self-management success.^23,24^ Disruptive family behaviors have been reported as major barriers to diabetes self-care in our recently completed mixed method study of 101 Chinese Americans with T2D.^25^

Family interventions involving both patients with T2D and family members may be particularly useful for Chinese Americans given that the Asian culture places a strong emphasis on family ties.^19,26,27^ When one family member is affected by a disease, the other family members are often willing to provide help and support.^27–29^ Family members are well-positioned to provide the long-term support that sustains beyond an intervention study. ^27–29^ Yet, family members often feel frustrated because they wanted to be involved in their loved ones’ diabetes care but lacked knowledge and skills necessary to be more supportive or helpful.^20,23^ Despite the significant role of family members in diabetes self-management success, most interventions reported in the literature failed to include family members.^19,30^

Mobile health (mHealth) such as using short message services (SMS) is a promising and practical strategy to deliver such a family intervention.^31,32^ A SMS-based family intervention is particularly relevant to Chinese Americans given that they often report long working hours and various challenges to attending in-person intervention sessions, which can be avoided using an mHealth approach.^16,28,29^ In addition, Chinese Americans are quite familiar with the SMS because of their social needs to connect with family and friends in the US and China.^25^ A SMS-based intervention has several advantages for educating and engaging patients in self-management and enhancing self-management support of their family members. In our recently completed mixed method study of 101 Chinese American patients with T2D, the idea of a SMS-based intervention was endorsed by the majority of participants.^25^ Participants also reported that their family members would be interested in receiving such an intervention.^25^ The efficacy of a family-based mHealth intervention involving both T2D patients and family members remains untested in Chinese Americans. To address this significant knowledge gap, the proposed study will leverage our current K99/R00 project, which aims to examine the potential efficacy of a patient-focused SMS intervention in Chinese Americans with T2D. This proposed study will adapt our K99/R00 **patient-focused** SMS intervention to a **family-oriented** SMS intervention, and pilot test it in a cohort of 30 Chinese American families (30 T2D patients and 30 their corresponding family members). Similar to the approach in our K99/R00 project, the culturally and linguistically adapted **family-oriented intervention will be delivered to participants via brief counseling videos sent to participants via SMS links (hereafter SMS intervention)**. Specifically, our aims are as follows:

## 2.2 Aims

**Primary Aim 1:** Assess the feasibility and acceptability of the SMS intervention in Chinese Americans with T2D and their family members

**Primary Aim 2:** Establish proof-of-concept regarding the potential efficacy of the SMS intervention for improving glycemic control

**Exploratory Aim:** Explore mediators of the SMS intervention

# 3. METHODS and procedures

## 3.1 Study Design

The proposed study is a pilot randomized controlled trial (RCT) of 6 months duration, with measurements at baseline, 3, and 6 months. Participants will be randomized to one of 2 arms (n=15 pairs each): 1) wait-list control; or 2) SMS intervention.

## 3.2 Sites

The study will be conducted at the Charles B. Wang Community Health Center (CBWCHC), a federally qualified health center located in both Manhattan Chinatown and Queens Flushing Chinatown. In 2015, it provided a total of 275,749 service visits to 50,008 underserved Asian Americans in Greater NYC. The majority of patients are Chinese. CBWCHC is one of the largest and leading community centers in NYC, having established a trusting relationship with the Chinese American community. In 2016, approximately 5,000 patients with T2D were seen at the CBWCHC; the vast majority of them are Chinese. CBWCHC will not be engaged in research activities and their role is to identify/refer patients and provide space for this study. All research related activities (e.g. recruitment, consenting, conducting study procedures) will be performed by NYU study staff. The letter of support has been uploaded in the attachment section.

To maximize recruitment efforts, we will also identify potential private practices or community based organizations in Chinatown areas and work with them to recruit potential participants. In addition, we will identify and recruit participants into the study from NYU Langone Health. **Similar to the procedures at CBWCHC, all of the research related activities (e.g. recruitment, consenting, conducting study procedures) will be performed by NYU study staff. The role of these private practices, NYU Langone Health, or community based organizations is to help to identify/refer patients and/or provide space for this study.**

## 3.3. Characteristics of the Research Population

We will recruit **60 participants for this project**, including 30 T2D Chinese patients and 30 corresponding family members. Family member is broadly defined as any support person who the patient considers plays a vital role in their diabetes self-management and would be interested in receiving such interventions. This can be spouse, adult children, siblings, or friends.

**Inclusion criteria for patient participants**: To be eligible for the study, patient participants must: 1) self-identify as Chinese immigrant or Chinese American; 2) be between the ages of 18 and 70; 3) be able to speak and understand Mandarin; 4) self-report a diagnosis of T2D; 5) baseline HbA1c ≥ 7%; 6) be currently using WeChat; 7) be willing to receive WeChat or text messages regarding T2D management; 8) express strong interest and confidence in finishing watching 2 diabetes videos each week for a total of 12 weeks; 9) be motivated to make lifestyle changes to control their diabetes; and 10) have a family member or friend be willing to participate in the study to learn about T2D to better support them.

**Inclusion criteria for family/friend participants**: Family/friend participants must 1) self-identify as Chinese immigrant or Chinese American; 2) be between 18-70 years old; 3) be able to speak and understand Mandarin; 4) be currently using WeChat or text messages; 5) be willing to receive WeChat or text messages regarding T2D management and learn how to better support the patient with T2D; 6) express strong interest and confidence in finishing watching 2 diabetes videos each week for a total of 12 weeks; and 7) be motivated to support their family/friend to make lifestyle changes to control their diabetes.

**Exclusion criteria for both patient and family/friend participants**: Individuals will be excluded from participation if they meet any of the following:(1) unable or unwilling to provide verbal consent; (2) unable to participate meaningfully in the intervention (e.g., uncorrected sight and hearing impairment); (3) unwilling to accept randomization assignment; (4) pregnant, plans to becomes pregnant in the next 6 months, or who become pregnant during the study, (5) breastfeeding, or (6) live in a facility or other health care setting where they have no control over diabetes self-management.

# 4. Procedures

## 4.1 Patient Recruitment

Participants be identified and recruited into the study from NYC health care facilities, including CBWCHC, private practices, and NYU Langone Brooklyn and affiliated provider practices.

We will recruit participants using the following methods**:**

**a. Posters.** Posters will be placed in NYC health care facilities waiting and examination rooms. Posters will list a contact telephone number that patients can call if interested in enrolling. Patients who self-refer will be screened for eligibility. Posters will also be placed in Chinese community centers (e.g., senior centers) and distributed during Chinese community events. Posters will also be posted electronically on WeChat, which is a very popular chatting smartphone application among Chinese Americans and Chinese Immigrants.

**b. Direct referral by NYC health care providers.** CBWCHC, NYU Langone Brooklyn site providers, and private practice health care providers will approach patients who are potentially eligible for the study, solicit their interest in the study. If patients express verbal interest in the study participation, their health care providers will share the patient’s contact information to the study staff, or patients will also be able to call (self-refer to) to the number provided in the recruitment flyer and speak with a study staff if they are interested in participating. Study staff will call the patient and screen for eligibility. Meanwhile, patients will be invited to call (self-refer to) the study staff if they are interested in participating. Study staff will call the patient and with the patient’s verbal consent the staff will conduct a quick phone pre-screening to confirm eligibility prior to enrollment. Dr. Qiuqu Zhao is the medical director at NYU Brooklyn Family Health Center. We are collaborating with Dr. Zhao and her team at NYU Brooklyn. They would help refer potential study participants that are eligible and interested in our study. The initial referral list from health care providers will only include patient’s name and phone number for those verbally expressed interest in the study. After verbal consent is obtained and documented, the study staff will request the patient’s medical information from the medical record.

**c. Registry lists**. From our previous study, we have created a registry list of potential participants who indicated interest in future studies from NYU School of Medicine. We will call participants from the registry list, explain the study, and assess the eligibility to participate.

**d. EPIC and DataCore.** We will work with DataCore to generate a report to identify potentially eligible subjects across NYU Langone Manhattan, Brooklyn, and Long Island campuses. Using a query, search will be conducted in EPIC to identify Chinese patients aged 18-70 and with type 2 diabetes whose most recent HbA1c value ≥ 7%. The report will also include demographics including patient name, gender/sex, date of birth, address, phone number, weight, height, BMI, name of primary care physician, most recent HbA1c value and date of measurement. Only the principal investigator and all members of the research team will have access to this report. The report will be generated prior to the recruitment process to help us find potential participants. The report will be kept on a secure electronic drive to ensure the confidentiality of health information and identifying information from potential participants. These patients will receive a mailing describing the study and letting them know how they can opt out of further contact. The study staff will call patients who have not opted out to describe the study, complete the brief eligibility screener and offer enrollment. While screening potential participants by telephone, demographics and the most recent HbA1c value will be confirmed or updated.

## 4.2 Patient Consent

The NYU study staff will be recruiting and consenting the participant by reading the approved telephone verbal informed consent script to the participant. The study staff will first complete a brief phone screener to screen whether or not the participant is eligible. Once eligibility is confirmed, the study staff will discuss with the patient what active participation in this study means (review verbal consent, interviews, potential benefits, and risks of participations), the purpose and limitations of the study, and requirements of the study. If the patients verbally agree to participate, the patient’s primary care physician will also be notified that the patient is being enrolled in this study. The verbal consent will be audiotaped with the patient’s permission. The verbal consent is an accommodation due to the rising COVID-19 infection rate in New York, and that the target population for this study is low-income aging immigrants with limited education and low digital literacy. All interventions will be conducted via phone or text message. A waiver of documentation of consent has been requested.

The study team has requested a waiver of signed HIPAA authorization and all HIPAA information will be presented verbally.

An IRB-approved verbal consent script will be used and will include the HIPAA and audio recording information, the subject will verbalize comprehension of the consent form and study enrollment. The consent process will be recorded via audio the consent and the date of consent will be documented on the Enrolled Participants Log. The Enrolled Participants Log is the only record linking a participant’s name and Subject ID, this information will be kept on a secure electronic drive to ensure the confidentiality of health information and identifying information from participants. Only the principal investigator and all members of the research team will have access to this information and the audio recordings of their verbal consent.

We will have the verbal consent script available in both English and Chinese. The participant can choose their own preferred language and our bilingual study staff will explain the study to the participant. The participant will be encouraged to ask any questions they may have before providing the verbal consent.

After verbal consent is obtained, we will ask both patients and family/friend participants if we can send them via text message a copy of the key information sheet for their records before we collect any data from them.

The Informed Consent is a continuous and dynamic process, all participants will be reminded at every phone call about the A/V recordings, study assessments and that they are free to withdraw at any time.

## 4.3 Study Procedures

The proposed study is a pilot randomized controlled trial (RCT) of 6 months duration. Participants will be randomized to one of 2 arms (n=15 pairs each): 1) wait-list control; or 2) SMS intervention. The study will include 3 telephone-based measurement visits, baseline, 3, and 6 months. For patient participant, during each telephone visit, we will ask them some survey questionnaires. Once we receive their consent, we will also contact their doctors’ office to obtain HbA1c results. For family/friend participant, each telephone visit will only have some survey questionnaires. At the end of the 3-month intervention, we will randomly select 10-20 patient and family member participants to join a focus group/interview study to understand their experience with our intervention. Both patient and their family member will be paid $30 for each measurement visit.

Upon receiving the referral list (interested patient’s name and phone number) from the providers, the bilingual study staff will call the patient and provide more details about the study. If patients expressed interest in participation, the study staff will administer several screening questions over the phone. After eligibility is determined, we will ask patients to talk to their family members or friends about this study and confirmed their family/friends interests in participation. Once we received call back from patients confirming their own as well as their family members/friends’ interests’, we will schedule an appointment to obtain verbal consent from both and collect baseline data. The patient and family/friend pair will be randomized via a computer-generated randomization scheme with equal allocation to one of the 2 groups: 1) wait-list control; or 2) SMS.

**Independent variable: randomization arm**

**Wait-list control group: For patient participants,** they will continue to receive the standard of usual care for their T2D at the NYC health care facilities during the course of our study. For family/friend participants, they will continue to receive their routine care with their own doctors during the study. At the end of the study, the wait-list control group (both patient and family/friend participants) will be provided the opportunity to receive the counseling videos delivered to them via SMS links.

**SMS or WeChat intervention:** We will send 2-3 diabetes videos every week for 12 weeks (each video about 5-10 minutes in duration) to both patients and their family members via WeChat or regular text messages (SMS). We will contact participants mainly through phone calls from NYU Langone Study Phone. WeChat will be only used to send the health education videos. No other communication will occur via WeChat. Our study team will remind participants at the beginning of the study and during the bi-weekly phone calls about not sending any Protected Health Information via WeChat. In addition, our education videos do not contain any Protected Health Information and it will teach participants about diet, exercise, and stress management in general. We will ask participants’ permission to use WeChat platform and advise that sending messages over an unsecured application like WeChat is an unsecure and unencrypted form of communication and there is a potential risk of disclosing information to an individual who is not authorized to receive it (unauthorized disclosure).

We will also let participants know that they can still participate in the study if they decline to receive the health education videos on WeChat. As an alternative, we will send the education videos as regular text messages to their phones. They can decline at any time during the study to receive the videos on WeChat and then we will switch to regular text messages.

Patients and their family members will receive the same intervention videos, including basic information about T2D, importance of diabetes self-management at home, behavioral techniques, and family-oriented sessions. For participants who miss 3 consecutive videos, the study team will follow-up with a phone call to identify barriers to watching the video and remind them to review the video. We will also help patient participants to set goals for their diabetes management and then have biweekly phone call follow-ups to check in their progress toward the goal and problem solve any raised questions/concerns.

**Procedures:** The study will include 3 measurement telephone visits, baseline, 3, and 6 months. Each session will include audio/Video recordings if verbal consent from the study participants was obtained. Both patient and family/friend participants will be paid $30 for each telephone visit. All data will be collected during the telephone visit. For patient participants, each visit will involve completion of questionnaires. After obtaining patients’ consent, we will contact their doctors’ office to obtain HbA1c results. For family/friend participants, each visit will only involve completion of questionnaires. For family/friend participants in the intervention group, we will send them diabetes education videos to help them obtain more information about diabetes, which allows them to better support patients in their disease management. For participants randomized to the SMS intervention group, we will reimburse $5 each week to each participant for cell phone data usage if they finish viewing both of the sent videos for that week. Given that we will send videos over a total of 12 weeks, participants may expect to receive a range of $0-$60 (=$5*12) depending on the number of weeks they have completed the videos. We will also provide $60 to control participants if they finish all study related procedures at 3 months assessment.

At the end of 3-month intervention, we will host 2 focus group discussions to understand their experience with our study. One focus group will invite highly engaged participants, defined as who have watched at least 80% of the diabetes videos. The other focus group is with poorly engaged group, defined as participants who have watched less than 80% of the videos. Each participant will receive $30 for their participation in the focus group. If it is challenging to identify a time that works for everyone, we will offer individual interview to those who have limited availability. After finishing the interview, they will also receive $30 as incentives.

**Study Variables**

**covariates**

**Behavioral changes**

- Diabetes self-management behaviors
- Dietary intake
- Physical activity

**Glycemic control**

**(HbA1c)**

**Interventions:**

- Wait-list control
- SMS

**Self-efficacy**

**Figure 1. Conceptual Model**

**Primary & Exploratory Outcomes**

**Primary outcome: Feasibility:** Feasibility will be examined by calculating the percent of videos sent that are watched. We will also assess the feasibility by determining whether we are able to meet our recruitment goal of 30 Chinese American families and reach 80% retention rate.

**Acceptability:** Acceptability will be assessed using a satisfaction questionnaire adapted from a prior SMS intervention study.^33,34^

**Secondary outcomes:** Consistent with our conceptual model (Fig. 1), we will also measure HbA1c, self-efficacy, and diabetes self-management behaviors at each time points (baseline, 3-, and 6-months). All of these scales have been used and validated in the Chinese population.

1. **HbA1c:** As part of the usual care, patients with T2D receive a HbA1c blood test at their doctors’ office every 3-6 months. To minimize participants’ burden, we will not conduct additional blood tests for this study. Instead, we will abstract the HbA1c testing results from the medical record at the participant’s health care facility. Once we obtain the consent from participants, we will contact their doctors’ offices for HbA1c results.
2. **Self-efficacy:** We will use the well-validated Stanford Diabetes Self-Efficacy Scale^36^ to measure participants’ confidence to manage T2D. This instrument contains 8 items and asks participants to rate their confidence level in performing specific self-management behaviors, using 10-point Likert scale ranging from 1 (not at all confident) to 10 (totally confident).
3. **Diabetes Self-Management Behaviors:** We will administer the adapted ﻿Summary of Diabetes Self-Care Activities (SDSCA) measure^37^ to assess participants’ adherence to diabetes self-management behaviors. This scale consists of 13 items and asks participate to describe their diabetes self-care activities over the past 7 days.
4. **Dietary Intake:** Informed by a previous study in Chinese Americans,^38^ we will use the adapted Mediterranean Dietary Screener^39^ to estimate dietary intake behaviors in our participants at baseline, 3- and 6-months.
5. **Physical Activity:** We will use the International Physical Activity Questionnaire (IPAQ)^42,43^ short version to assess the frequency and duration of various physical activities undertaken by adults over the past 7 days. This scale will provide an estimate of the number of minutes per week participants engage in each category of physical activity (e.g., vigorous, moderate, and mild intensity).

**Covariates: Sociodemographic, health characteristics, and acculturation.** We will use a sociodemographic questionnaire to collect basic information about the participant such as age, gender, education, income, duration of residence in the US, health insurance, the diabetes medication regimen, duration of T2D, and medical history. Informed by prior literature, we will also include surveys to measure Health Beliefs, Diabetes Knowledge, Diabetes Distress, Social Support, Mental Health, the Impact of COVID-19, and memory test, which also play essential roles in diabetes management.

#

# 5. Risk/Benefit Assessment

## 5.1 Potential Risks

Risks of participation in this study are minimal and unlikely to occur given the safeguards in place. Psychological risks include: 1) the possibility that some participants may perceive some questionnaire or background data assessment as intrusive or causing them to feel uncomfortable; 2) breach of confidentiality. For the confidentiality protection related to audio-recording, we will store all the recordings on a password protected server. We will use a unique code number during the transcription. Once the transcription is done, all the audio recordings will be destroyed.

There is a potential risk for breach of confidentiality on WeChat. To minimize the risk, we will remind participants that they should call our study phone number for any questions related to the study participation. WeChat will only be used to send education videos. No communication will occur via WeChat. Also, we will remind them at the beginning of the study and during the bi-weekly phone calls about not sending any Protected Health Information via WeChat. In addition, our education videos do not contain any Protected Health Information and it will teach participants about diet, exercise, and stress management in general. Based on our recent completed pilot study (a similar WeChat video study in 30 Chinese immigrants), we did not observe any breach of privacy or confidentiality. All of the study team members received sufficient training on protecting privacy and confidentiality and have extensive experience in working with this hard-to-reach immigrant population. The benefits of receiving these culturally and linguistically tailored education videos outweighed the risks.

The study involves an intervention designed to enhance adherence to standard diabetes care (e.g., adherence to the prescribed diabetes medication, healthy eating, and physical activity behaviors). If our intervention improves adherence to the medication regimen or if participants make lifestyle changes (e.g., increased physical activity), more episodes of hypoglycemia may result. We will advise participants of the importance of monitoring their blood sugar as suggested by their doctors, and to contact their doctors as soon as they experience low blood sugar.

## 5.2 Benefits

We do not anticipate that participants will receive any direct benefit from participating. However, information learned during the study may produce benefits for future patients by improving their diabetes care.

# 6. Confidentiality

We will use the IRB recommended RedCap for data storage (openREDCap.nyumc.org). We will also work with MCIT to set up a NYU shared network drive which is HIPAA compliant (for offsite backup storage) specifically for our study. All study personnel have already taken the mandatory HIPAA and Patient Privacy/Confidentiality training modules required by our institutional IRBs, to ensure that they are aware of the importance of patient confidentiality and all appropriate laws regarding protection against privacy breaches. Procedures will be in place to ensure that all files containing subject information will be kept in locked filing cabinets or password-protected electronic databases on a secure server. The majority of study data and all interview transcripts will be maintained in electronic files with no patient identifiers other than a study ID number; there will be one file maintained separately with its own password protection that links study ID numbers to patient identifying information.

No identifiable information about study participants will be disclosed to individuals outside those approved by the IRB to be on the study protocol. No publications that result from the study will identify individual participants.

Transcript files will also be password-protected and user-restricted. We will delete potential identifiable information from transcripts and audio/video recordings one year after the study ends. We will also encourage participants to use pseudonyms during focus groups and/or interviews. During each focus group session, the study PI will emphasize the importance of privacy and confidentiality of the discussion within the group. The text-message-based video counseling will be protected via password-enabled access. Only study participants will have access codes to open these videos. Others in the same household would not be able to open the video, unless participants choose to share the access code.

# 7. Data Analysis and Data Monitoring

## 7.1 Data Analysis

**Data analysis for Aim 1. Feasibility and acceptability**: Feasibility will be examined by calculating the percent of videos sent that are watched. We will also report the actual enrollment and retention rate. Acceptability will be examined by calculating percent of participants who report satisfaction with our intervention using a questionnaire adapted from a prior SMS intervention study.^33,34^

**Data analysis for Aim 2.** We will employ the “intent to treat” (ITT) approach in analyses. We will perform a detailed descriptive analysis of all the data collected in the study. These preliminary descriptive statistics will be used to 1) check accuracy and completeness of inputted data, 2) describe univariate distribution of each variable at baseline, and 3) examine the associations between variables. We will also explore features of the data (e.g., amount and pattern of missing data, outliers, excess zeros, departures from distributional assumptions) to determine whether special techniques are needed.

**Aim 2: Proof of concept regarding the potential efficacy of the intervention for glycemic control.** We will test whether the 2 groups are comparable on baseline sociodemographic, health characteristics, and levels of acculturation. If significant differences are found, we will include the variables as covariates in the models. Between group and group by time interaction effects will be examined graphically and via linear mixed modeling for HbA1c. Calculated effect sizes will be used for sample size estimation needed for a two-sided alpha of 0.05 and power of 0.80 for the larger scale R01 study.

**Exploratory Aim: Explore the mediators of the SMS intervention.** Although underpowered, we will perform exploratory analyses of candidate mediators to evaluate the merit of further testing in a larger, adequately powered study. We will perform mediation analyses to determine whether self-efficacy and adherence to diabetes self-management explain significant variance in improvements in HbA1c.

**Focus group**: All focus groups will be audio-recorded and transcribed verbatim into Microsoft Word. Atlas will be used to store, organize, retrieve, and analyze the qualitative data. Thematic analysis will be used to analyze qualitative data.^41^ To establish coding reliability, the RA and PI will independently code up to 10% of the transcripts and identify kappa coefficient.^42^ We will meet weekly to review definitions and assignment of codes, and resolve differences through consensus. If no agreement can be made, the study team will be consulted. The RA and PI will read all transcripts and develop an open coding schema based on emergent theses. We will present coding schemes to study team, discuss emergent themes, refine codes, and develop a codebook that consists of a list of categories and topics. Once all data have been coded based on the codebook, codes most relevant to the research questions will be subject to further analysis.

## 7.2 Data Monitoring

The purpose of the data safety monitoring plan is to ensure the safety of subjects and the validity and integrity of the data. Data and safety monitoring will be the shared responsibility of all members of the research team. Personnel involved in monitoring activities will include:

- Dr. Mary Ann Sevick, ScD (co-investigator). Professor of Population Health at NYU School of Medicine (NYUSoM).
- Dr. Lu Hu, PhD, PI, Dr. Hu has worked as a graduate student researcher on several NIH-funded projects during her doctoral training and participated in the data and safety monitoring meetings. During her current postdoctoral training, she is also actively participating in Dr. Sevick’s project meetings, including data and safety monitoring meetings. Dr. Hu has received intensive training in human subjects research. Dr. Hu will be responsible for data safety monitoring of the overall study and Dr. Sevick will provide close oversight via weekly mentoring meeting.

**Data monitoring**

Ongoing quality control will include regular data verification and protocol compliance checks. An ongoing review of study procedures will be done to ensure that the privacy of subjects and confidentiality of data is not violated. Weekly meetings will be held between Dr. Hu and Dr. Sevick and other research staff involved in the project to review the progress of subjects enrolled in the study.

**Safety monitoring**

It is possible that our intervention might help participants better adhere to the medication regimen or make lifestyle changes (e.g., increased physical activity), which may result in the need for less diabetes medications and thus more episodes of hypoglycemia might happen. We will advise them to continue to monitor their blood sugar levels as suggested by their doctors, and contact their doctors if they experience any low blood sugar levels. This safety information will be emphasized at the beginning and end of each intervention video.

Because the proposed study is minimal risk, we will not establish an independent Data Safety and Monitoring Board. The study team including several experts (Drs. Sevick, Hu) who have extensive experience in conducting clinical trials will perform date safety and monitoring. We will meet regularly to review inclusion and exclusion criteria, and develop a detailed safety protocol, plans for monitoring subjects for adverse events, and a protocol for protection of privacy and confidentiality of participants. The study PI (Dr. Hu) will hold weekly project meeting with the study staff and provide project updates to Dr. Sevick (primary mentor) via weekly mentoring one-on-one meetings. The study PI will provide updates on the study progress, recruitment, retention, data quality, safety, confidentiality, and the occurrence of adverse events to the whole mentoring team during bi-monthly check-in meetings.

The whole team will provide oversight for the following study procedures:

- Monitoring the safety of the subjects (e.g., review the research protocol and plans for data and safety monitoring)
- Evaluate the progress of the clinical trial, including periodic assessments of data quality and timeliness, subject recruitment, accrual and retention, subjects risk versus benefit, performance of the recruitment site, and other factors that can affect study outcome
- Maintaining the confidentiality and integrity of the data
- Reports of critical or adverse events from research staff. The PI will receive these reports on an event-by-event basis and will inform the whole study team of all such reports
- Make recommendations to the IRB and investigators concerning continuation or conclusion of the trial.

_

# 8. Investigators Qualifications

Mary Ann Sevick, ScD (diabetes mHealth behavioral expert) is a Professor at the NYU School of Medicine. Dr. Sevick has over 25 years of experience in conducting large, theory-based behavioral intervention trials. She has been the PI/Co-I on multiple NIH and foundational grants (R01-NR008792; R01-NR010135; K24- NR012226; HSR&D-IIR07154; AHA-17SFRN33590133). She is currently the PI of an R01 study examining technology-based behavioral interventions in patients with T2D and concurrent kidney disease. She is also the PI of an R21 study examining the use of video-based behavioral counseling interventions in hemodialysis patients and the PI of an American Heart Association (AHA) Obesity Network Center study investigating a novel, personalized dietary weight-loss approach in patients with prediabetes. Dr. Sevick has mentored 10 junior faculty members and 18 postdoctoral fellows. Given her extensive expertise, she will serve as a content expert in T2D, theory-based behavioral intervention, mHealth, and academic career mentor.

Lu Hu, PhD, is well poised to serve as the co-investigator on the proposed study based on 1) her rigorous doctoral training in chronic disease management and postdoctoral and 2016 NIMHD Summer training in health disparity, 2) her current role as a co-investigator on an Aetna-funded study involving the use of a mHealth intervention (e.g., text messages) to remotely titrate the insulin regimen in underserved low-income patients with uncontrolled type 2 diabetes (T2D), 3) a strong publication record of about 20 published/accepted papers, and 4) several national and international awards and fellowships.

All investigator’s CV and medical licenses are attached to the protocol submission. All investigators and staff involved in this project have completed training on the protection of human subjects in research through CITI training and HIPAA certification.

# 9. Study Records Retention and Data Sharing

We are firmly committed to sharing data with the scientific community so that the data generated from this study can be fully utilized for research. Provided below is the proposed data-sharing plan:

1. We will publish the detailed methodology used for this study. Aggregated statistics will be provided to the broad scientific community via a journal’s website or to an individual investigator/team upon request.

2. We will release the data generated from all study participants to qualified researchers who wish to collaborate with the investigators from our study. Investigators who wish to collaborate should submit a proposal that will be reviewed using criteria similar to those used by the NIH \for scientific merit and human subject protection. All data should be used for research only. No data will be provided that could potentially disclose the identities of study participants.

NIH may release new policies regarding data sharing during the study period. We will be willing to discuss such policies with NIMHD program officers and modify the data-sharing plan detailed above.

# 10. References

1. Zong J, Batalova J. Chinese Immigrants in the United States | migrationpolicy.org. Migration policy institute. http://www.migrationpolicy.org/article/chinese-immigrants-united-states. Published 2017. Accessed May 30, 2017.

2. Passel JS, Cohn D. U.S. Population Projections: 2005-2050 |. Pew Research Center. http://www.pewsocialtrends.org/2008/02/11/us-population-projections-2005-2050/. Published 2008. Accessed May 30, 2017.

3. Hoeffel EM, Rastogi S, Kim MO, Shahid H. The Asian Population: 2010 2010 Census Briefs. 2012. https://www.census.gov/prod/cen2010/briefs/c2010br-11.pdf. Accessed May 30, 2017.

4. McNeely MJ, Boyko EJ. Type 2 diabetes prevalence in Asian Americans: results of a national health survey. *Diabetes Care*. 2004;27(1):66-69. http://www.ncbi.nlm.nih.gov/pubmed/14693968. Accessed May 30, 2017.

5. Menke A, Casagrande S, Geiss L, Cowie CC. Prevalence of and Trends in Diabetes Among Adults in the United States, 1988-2012. *JAMA*. 2015;314(10):1021. doi:10.1001/jama.2015.10029

6. Islam NS, Wyatt LC, Kapadia SB, Rey MJ, Trinh-Shevrin C, Kwon SC. Diabetes and associated risk factors among Asian American subgroups in New York City. *Diabetes Care*. 2013;36(1):2013. doi:10.2337/dc12-1252

7. Thorpe LE, Upadhyay UD, Chamany S, et al. Prevalence and control of diabetes and impaired fasting glucose in New York City. *Diabetes Care*. 2009;32(1):57-62. doi:10.2337/dc08-0727

8. Rajpathak SN, Wylie-Rosett J. High prevalence of diabetes and impaired fasting glucose among chinese immigrants in New York City. *J Immigr Minor Heal*. 2011;13(1):181-183. doi:10.1007/s10903-010-9356-2

9. Ma RCW, Chan JCN. Type 2 diabetes in East Asians: similarities and differences with populations in Europe and the United States. *Ann N Y Acad Sci*. 2013;1281(1):64-91. doi:10.1111/nyas.12098

10. Brown AF, Gregg EW, Stevens MR, et al. Race, ethnicity, socioeconomic position, and quality of care for adults with diabetes enrolled in managed care: the Translating Research Into Action for Diabetes (TRIAD) study. *Diabetes Care*. 2005;28(12):2864-2870. doi:10.2337/DIACARE.28.12.2864

11. Herman WH, Dungan KM, Wolffenbuttel BHR, et al. Racial and ethnic differences in mean plasma glucose, hemoglobin A1c, and 1,5-anhydroglucitol in over 2000 patients with type 2 diabetes. *J Clin Endocrinol Metab*. 2009;94(5):1689-1694. doi:10.1210/jc.2008-1940

12. Xu Y, Pan W, Liu H. Self-management practices of Chinese Americans with type 2 diabetes. *Nurs Heal Sci*. 2010;12(2):228-234. doi:10.1111/j.1442-2018.2010.00524.x

13. Islam NS, Kwon SC, Wyatt LC, et al. Disparities in diabetes management in Asian Americans in New York City compared with other racial/ethnic minority groups. *Am J Public Health*. 2015;105:S443-S446. doi:10.2105/AJPH.2014.302523

14. Lanting LC, Joung IMA, Mackenbach JP, Lamberts SWJ, Bootsma AH. Ethnic differences in mortality, end-stage complications, and quality of care among diabetic patients: a review. *Diabetes Care*. 2005;28(9):2280-2288. http://www.ncbi.nlm.nih.gov/pubmed/16123507. Accessed May 30, 2017.

15. Tseng J, Halperin L, Ritholz MD, Hsu WC. Perceptions and management of psychosocial factors affecting type 2 diabetes mellitus in Chinese Americans. *J Diabetes Complications*. 2013;27(4):383-390. doi:10.1016/j.jdiacomp.2013.01.001

16. Chun KM, Chesla CA, Kwan CML. “So We Adapt Step by Step”: Acculturation experiences affecting diabetes management and perceived health for Chinese American immigrants. *Soc Sci Med*. 2011;72(2):256-264. doi:10.1016/j.socscimed.2010.11.010

17. Ho EY, Tran H, Chesla C a. Assessing the Cultural in Culturally Sensitive Printed Patient-Education Materials for Chinese Americans With Type 2 Diabetes. *Health Commun*. 2014;0236(February):37-41. doi:10.1080/10410236.2013.835216

18. King GL, Mcneely MJ, Thorpe LE, et al. Understanding and addressing unique needs of diabetes in Asian Americans, Native Hawaiians, and Pacific Islanders. *Diabetes Care*. 2012;35(5):1181-1188. doi:10.2337/dc12-0210

19. Zeng B, Sun W, Gary RA, Li C, Liu T. Towards a conceptual model of diabetes self-management among Chinese immigrants in the United States. *Int J Environ Res Public Health*. 2014;11(7):6727-6742. doi:10.3390/ijerph110706727

20. Mayberry LS, Harper KJ, Osborn CY. Family behaviors and type 2 diabetes: What to target and how to address in interventions for adults with low socioeconomic status. *Chronic Illn*. 2016;12(3):199-215. doi:10.1177/1742395316644303

21. A.a. B, A. B, M.T. Q. Family interventions to improve diabetes outcomes for adults. *Ann N Y Acad Sci*. 2015;1353(1):89-112. doi:10.1111/nyas.12844.Family

22. Polly R, Sawin K. The Individual and Family Self-management Theory: *Nurs Outlook*. 2009;57(4):217-225. doi:10.1016/j.outlook.2008.10.004.The

23. Mayberry LS, Osborn CY. Family involvement is helpful and harmful to patients’ self-care and glycemic control. *Patient Educ Couns*. 2014;97(3):418-425. doi:10.1016/j.pec.2014.09.011

24. Mayberry LS, Osborn CY. Family Support, Medication Adherence, and Glycemic Control Among Adults With Type 2 Diabetes. *Diabetes Care*. 2012;35(6):1239-1245. doi:10.2337/dc11-2103

25. Hu L, Trinh-Shevrin C, Yi SS, et al. Diabetes Management and Technology Use among Chinese Americans in NYC: Current Status and Future Opportunities. In: Poster Presentation at the 9th Biennial Asian American, Native Hawaiian, and Pacific Islander Health Conference, New York, NY; 2018.

26. Chesla C a, Chun KM. Accommodating type 2 diabetes in the Chinese American family. *Qual Health Res*. 2005;15(2):240-255. doi:10.1177/1049732304272050

27. Chesla CA, Kwan CML, Chun KM, Stryker L. Gender Differences in Factors Related to Diabetes Management in Chinese American Immigrants. *West J Nurs Res*. 2014;36(9):1074-1090. doi:10.1177/0193945914522718

28. Chun KM, Chesla † CA. Cultural issues in disease management for chinese americans with type 2 diabetes. *Psychol Health*. 2004;19(6):767-785. doi:10.1080/08870440410001722958

29. Chesla CA, Chun KM, Kwan CML. Cultural and family challenges to managing type 2 diabetes in immigrant Chinese Americans. *Diabetes Care*. 2009;32(10):1812-1816. doi:10.2337/dc09-0278

30. Mayberry LS, Berg CA, Harper KJ, Osborn CY. The Design, Usability, and Feasibility of a Family-Focused Diabetes Self-Care Support mHealth Intervention for Diverse, Low-Income Adults with Type 2 Diabetes. *J Diabetes Res*. 2016;2016. doi:10.1155/2016/7586385

31. de Jongh T, Gurol-Urganci I, Vodopivec-Jamsek V, Car J, Atun R. Mobile phone messaging for facilitating self-management of long-term illnesses. *Cochrane database Syst Rev*. 2012;12(12):CD007459. doi:10.1002/14651858.CD007459.pub2.Copyright

32. T DJ, Atun R, Car J. Mobile phone messaging for preventive health care ( Review ). 2012;(12). doi:10.1002/14651858.CD007457.pub2.www.cochranelibrary.com

33. Nundy S, Dick JJ, Chou C-H, Nocon RS, Chin MH, Peek ME. Mobile Phone Diabetes Project Led To Improved Glycemic Control And Net Savings For Chicago Plan Participants. *Health Aff*. 2014;33(2):265-272. doi:10.1377/hlthaff.2013.0589

34. Dick JJ, Nundy S, Solomon MC, Bishop KN, Chin MH, Peek ME. Feasibility and usability of a text message-based program for diabetes self-management in an urban African-American population. *J Diabetes Sci Technol*. 2011;5(5):1246-1254. doi:10.1177/193229681100500534

35. Bijl J V, Poelgeest-Eeltink A V, Shortridge-Baggett L. The psychometric properties of the diabetes management self-efficacy scale for patients with type 2 diabetes mellitus. *J Adv Nurs*. 1999;30(2):352-359. http://www.ncbi.nlm.nih.gov/pubmed/10457237. Accessed August 23, 2017.

36. Pace AE, Gomes LC, Bertolin DC, Loureiro HMAM, Bijl J Van Der, Shortridge-Baggett LM. Adaptation and validation of the Diabetes Management Self-Efficacy Scale to Brazilian Portuguese. *Rev Lat Am Enfermagem*. 2017;25(0). doi:10.1590/1518-8345.1543.2861

37. Lee E-H, van der Bijl J, Shortridge-Baggett LM, Han SJ, Moon SH. Psychometric Properties of the Diabetes Management Self-Efficacy Scale in Korean Patients with Type 2 Diabetes. *Int J Endocrinol*. 2015;2015:780701. doi:10.1155/2015/780701

38. Schmitt A, Gahr A, Hermanns N, Kulzer B, Huber J, Haak T. The Diabetes Self-Management Questionnaire (DSMQ): development and evaluation of an instrument to assess diabetes self-care activities associated with glycaemic control. *Health Qual Life Outcomes*. 2013;11:138. doi:10.1186/1477-7525-11-138

39. Schmitt A, Reimer A, Hermanns N, et al. Assessing Diabetes Self-Management with the Diabetes Self-Management Questionnaire (DSMQ) Can Help Analyse Behavioural Problems Related to Reduced Glycaemic Control. Wang X, ed. *PLoS One*. 2016;11(3):e0150774. doi:10.1371/journal.pone.0150774

40. Stephenson M. Development and Validation of the Stephenson Multigroup Acculturation Scale (SMAS). *Psychological*. 2000;12(I):77-88. doi:10.1037//1040-3590.12.1.77

41. Fereday J, Muir-Cochrane E. Demonstrating Rigor Using Thematic Analysis: A Hybrid Approach of Inductive and Deductive Coding and Theme Development. *Int J Qual Methods*. 2006;5(1):80-92. doi:10.1177/160940690600500107

42. Burla L, Knierim B, Barth J, Liewald K, Duetz M, Abel T. From Text to Codings. *Nurs Res*. 2008;57(2):113-117. doi:10.1097/01.NNR.0000313482.33917.7d
