# Supplementary material for: Feasibility of a family-oriented mHealth intervention for Chinese Americans with type 2 diabetes: A pilot randomized control trial
Source: PLoS One. 2024 Mar 11;19(3):e0299799. doi: 10.1371/journal.pone.0299799 (PMC10927140; doi:10.1371/journal.pone.0299799)
Supplement: S4 File — (PDF) [file pone.0299799.s004.pdf]

Office of Science and Research
Institutional Review Board
1 Park Avenue | 6th Floor | New York, NY 10016

Approval of Submission

August 19, 2019

On 8/19/2019 the IRB reviewed and approved the following submission:

principal investigator	Mary Sevick
study number	i19-01275
study title	Feasibility of a Family-oriented mHealth Intervention for Chinese Americans with Type 2 Diabetes
performance period	8/19/2019
location(s)	Charles B Wang (Other Locations)
sponsor(s)	Name: NIH-NIDDK
review type	Initial Study, Expedited Category 2,5,6,7;
board name	All boards
materials approved for use	• Follow-up patient survey_08.12.2019.pdf, Category: IRB Protocol;<br>• Study poster_08.12.2019.pdf, Category: Recruitment Materials;<br>• ICF-Key-Information_08.15.2019.pdf, Category: Consent Form;<br>• Baseline patient survey_08.12.2019.pdf, Category: IRB Protocol;<br>• Follow-up family_friend survey_08.16.2019.pdf, Category: IRB Protocol;<br>• Baseline family_friend survey_08.12.2019.pdf, Category: IRB Protocol;<br>• AV consent_08.15.2019.pdf, Category: Consent Form;<br>• Consent form_08.15.2019.pdf, Category: Consent Form;<br>• Protocol_08.06.2019 (updated).pdf, Category: IRB Protocol;

The current IRB Status of your submission is: Approved. This submission was reviewed by the NYU School of Medicine's Institutional Review Board (IRB). During the review of your study, the IRB specifically considered:

1. the risks and anticipated benefits (if any) to your subjects
2. the selection of subjects
3. the procedures for securing and documenting informed consent
4. the safety of your subjects
5. the privacy of your subjects and confidentiality of the data

Your study cannot commence until all ancillary review decisions are complete. To determine the state of all ancillary reviews, go the MyStudies page of this study in Research Navigator. Ancillary review statuses are located on the top/right area of your study's main screen.

Note: Ensure that approval has been issued in MyAgreements/CRMS and the Clinical Research Support Unit ("CRSU") before you proceed with any aspect of this study, including the enrollment of human subjects.

Review Notes

For NIH grant funded research approved before the revised Common Rule: the IRB has found the IRB approved protocol referenced above to be consistent with the NIH grant application.

Sincerely,

8/19/2019

RE: Study# i19-01275

Helen Panageas
Director, NYU SoM Institutional Review Board
Federalwide Assurance: FWA00004952

NYU School of Medicine's IRB operates in accordance with Good Clinical Practices (GCP) and applicable laws and regulations. Federal rules allow IRBs to document their determination/authorization process in their policy manual. Determination letters generated by NYU SoM's IRB administration system are not physically signed as per policy. All approved study materials are clearly identified and locked in each study submission record within the IRB's administration system.

NYU School of Medicine IRB Policy

- *All current IRB policy documents can be found on our website*
- *You must submit all modifications to this study (e.g., protocol updates, modified recruitment materials, consent forms, etc.) using Research Navigator to communicate with the IRB ("eSubmission") for review and approval prior to initiation of those change(s), except where necessary to eliminate apparent immediate hazards to the subject(s). Changes made to eliminate apparent immediate hazards to subjects must be reported to the IRB within 24 hours.*
- *All adverse and/or unanticipated event(s) that occur while conducting this study must immediately be reported to the IRB via eSubmission.*
- *You may only use IRB-approved copies of your consent form(s), questionnaire(s), letter(s), advertisement(s), etc. in your study. Never use expired consent forms.*
- *If modifications are made to the study or adverse events occur while conducting study, the PI must inform all research staff listed on this study.*
- *IRB's approval is valid as per the period indicated above. A reminder to submit a continuation (should one be required) will be e-mailed to the PI, PI Proxy and Primary Contact 90, 60 and 30 days prior to this study's expiration date if one is indicated. After expiration, a daily reminder will be sent for 30 days followed by a weekly reminder until the study receives re-approval or a study closure.*
- *Prior to initiating an IRB-approved study, you must receive written approval from an authorized representative for each site where your study will take place. Key contacts are:*
  - Bellevue Hospital (BHC): if you are conducting all or part of your study at BHC, you must contact them to obtain additional approvals. BHC will be notified if any of their sites are selected as a location where your study takes place, but your team is obligated to contact them at BellevueResearch@bellevue.nychhc.org to find out what approvals are required before conducting any research at a BHC location.
  - *CTSI - Clinical and Translational Science Institute, NYU School of Medicine [formerly General Clinical Research Center (GCRC)]: email ctsi@nyulangone.org*
  - *NYU Langone Health Centers (Tisch Hospital/Rusk Institute/Co-op Care/HJD/Perlmutter Cancer Center) site approval is handled for you automatically (as needed) by the CRSU*
- *The IRB may suspend or terminate studies that are not in compliance with NYU Langone Health/School of Medicine Policies & Procedures and the requirements of the Institution's Federal Wide Assurance with the federal government.*
- *Direct IRB questions and comments to 212-263-4110 or IRB-INFO@nyulangone.org*

Let Us Know How We're Doing

Click on the title above to send us feedback via a short, anonymous survey. Providing exceptional customer service is a top priority of the IRB and your responses will help us understand how we can continue to improve our service to the research community.

Institutional Review Board
Human Research Protection Program
1 Park Avenue | 6th Floor | New York, NY 10016
http: – – irb.med.nyu.edu

19-AUG-2019

IRB Board Rosters

Effective **2019-08-06** FWA#00004952

Quorum is simple majority only = greater than half

Board A (Effective 2019-08-06)

Members; Quorum of 7; 8 for prisoner-related studies

Member	Degree(s)	Science – Non-Science	Specialty	Affiliated With NYU	Alternate(s)
More, Frederick (Chair)	DDS	S	Dentistry – Pediatrics	Y	Hazen, Katz, Nishawala, Novik, Wissner-Greene
Donnino, Robert (Vice Chair)	MD	S	Medicine – Cardiology	Y	Vice Chair Alternates: Hazen, Nishawala, GreeneMember Alternates: Culliford, Katz
Basu Roy, Upal	PhD, MPH	S	Community Representative	N	Abramovitz, Raskin, Wu
Bliss, Samuel	PharmD	S	Pharmacy	Y	Ballani, Dubrobskaya, Faiena, Wong
Diefenbach, Catherine	MD	S	Oncology	Y	Esteva, Kwa, Moskovitz, Novik, Rapp, Ryan, Saint Fleur, Schiff, Wu
Gallagher, Richard	PhD	S	Child & Adolescent Psychiatry	Y	Barr, Bogenschutz, Chervinsky, Frankle, McGregor, Nishawala, Rotrosen
Godina, Marina	RN	S	IRB Administration; Nursing	Y	Berkovitz, Jeschke-Lopez, Johnson
Kadidal, Shane *	JD	NS	Law – Prisoner Advocate	N	
Lamont, Justin	MD	S	Orthopedic Surgery	Y	Kirsch, Leucht, Rapp
Panageas, Helen	BA	NS	IRB Administration	Y	Dvorkin, Liu, McGowan, Vieira, Wallach
Ross-Rizzo, John	MD	S	Neurology	Y	Barr, Berk, Chervinsky, Lewis
Ross, Stephen	MD	S	Psychiatry	Y	Barr, Bogenschutz, Chervinsky, Frankle, McGregor, Nishawala, Rotrosen
Storey, Elizabeth	PhD	S	Radiology	Y	Bencardino, Fieremans, Gonen
Wang, Jing	MD	S	Anesthesiology	Y	Kim

Counts toward quorum only when reviewing studies subject to Subpart C

Board B (Effective 2019-08-06)

Members; Quorum of 9, 9 for prisoner-related studies

Member	Degree(s)	Science – Non-Science	Specialty	Affiliated With NYU	Alternate(s)
Katz, Stuart (Chairman)	MD PhD	S	Cardiology	Y	Donnino, Hazen, More, Nishawala, Novik, Wissner-Greene
Hazen, Alexes (Vice Chair)	MD	S	Surgery – Plastic	Y	Vice Chair Alternates: Donnino, More, Nishawala, Novik, Wissner-Greene Member Alternates: DeLacure
Bartlett, Rachel	PhD	S	Radiology	Y	Bencardino, Fieremans, Gonen, Storey
Berk, Thomas	MD	S	Neurology	Y	Barr, Lewis, Ross-Rizzo
Brar, Preneet	MD	S	Pediatric Endocrinology	Y	Ramirez, Tunik, Wissner-Greene
Culliford, Alfred	MD	S	Surgery – Cardiothoracic	Y	Donnino, Lamont, Rapp
Dubrovskaya, Yanina	PharmD	S	Pharmacy	Y	Ballani, Bliss, Faiena, Wong
Esteva, Francisco	MD	S	Oncology	Y	Diefenbach, Moskovits, Novik, Rapp, Ryan, Saint Fleur, Schiff, Wu
Frankle, William Gordon	MD	S	Psychiatry	Y	Barr, Bogenschutz, Gallagher, McGregor, Nishawala, Ross, Rotrosen
Godina, Marina	RN	S	IRB Administration; Nursing	Y	Berkovitz, Jeschke-Lopez Johnson
Kadidal, Shane *	JD	NS	Law-Prisoner Advocate	N	
Kirsch, Thorsten	PhD	S	Orthopedic Surgery	Y	Lamont, Leucht, Rapp
Kwa, Maryann	MD	S	Oncology	Y	Diefenbach, Moskovits, Novik, Rapp, Ryan, Saint Fleur, Schiff, Wu
McGowan, Richard	MLS	NS	Public Services	Y	Vieira
Nolan, Anna	MD	S	Pulmonary	Y	
Panageas, Helen	BA	NS	IRB Administration	Y	Dvorkin, Liu, Vieira, Wallach
Raskin, Joyce	JD	NS	Community Representative	Y	Abramovitz, Basu Roy, Wu

**Counts toward quorum only when reviewing studies subject to Subpart C*

19-AUG-2019 19-AUG-2019 19-AUG-2019

Board C (Effective 2019-08-06)

Members; Quorum of 6; 7 for prisoner-related studies

Member	Degree(s)	Science – Non-Science	Specialty	Affiliated With NYU	Alternate(s)
Novik, Yelena (Chair)	MD	S	Oncology	Y	Donnino, Hazen, Katz, More, Nishawala, Wissner-Greene
Nishawala, Melissa (Vice Chair)	MD	S	Child & Adolescent Psychiatry	Y	Vice Chair Alternates: Donnino, Hazen, Katz, More, Wissner-Greene<br>Member Alternates: Barr, Bogenschutz, Chervinsky, Frankle, Gallagher, McGregor, Ross, Rotrosen
Godina, Marina	RN	S	IRB Administration; Nursing	Y	Berkovitz, Jeschke-Lopez, Johnson
Chervinsky, Alexander B	PhD	S	Neuropsychology	Y	Barr, Bogenschutz, Gallagher, Lewis, McGregor, Nishawala, Ross, Rotrosen
Kadidal, Shane *	JD	NS	Law – Prisoner Advocate	N	
Kim, Sunmi	MD	S	Anesthesiology	Y	Wang
Panageas, Helen	BA	NS	IRB Administration	Y	Dvorkin, Liu, McGowan, Wallach
Schiff, Peter	MD	S	Radiation Oncology	Y	Diefenbach, Esteva, Kwa, Moskovits, Rya, Saint Fleur
Vieira, Dorice	MA	NS	Public Services	Y	McGowan
Wong, Doris	PharmD	S	Pharmacy	Y	Ballani, Bliss, Dubrovskaya, Faiena
Wu, Jennifer	MD	S	Oncology	Y	Diefenbach, Esteva, Kwa, Moskovits, Ryan, Saint Fleur
Wu, Lillian	BA, MA	NS	Community Representative	N	Abramovitz, Basu Roy, Raskin

**Counts toward quorum only when reviewing studies subject to Subpart C*

19-AUG-2019

Board D (Effective 2019-08-06)

Members; Quorum of 9; 9 for prisoner-related studies

Member	Degree(s)	Science – Non-Science	Specialty	Affiliated With NYU	Alternate(s)
More, Frederick (Chair)	DDS	S	Dentistry – Pediatrics	Y	Donnino, Hazen, Katz, Nishawala, Novik, Wissner-Greene
Wissner-Greene, Loren (Vice Chair)	MD, MA	S	Medicine – Endocrinology – Ob-Gyn	Y	Vice Chair Alternates: Donnino, Hazen, Katz, More, Nishawala, Novik, Member Alternate: Brar
Abramovitz, Rachel	LL.M., LL.B.	NS	Community Representative	N	Basu Roy, Raskin, Wu
Ballani, Kanika	PharmD	S	Pharmacy	Y	Bliss, Dubrovskaya, Faiena, Wong
Barr, William	PhD	S	Neuropsychology	Y	Berk, Chervinsky, Frankle, Gallagher, Lewis, McGregor, Nishawala, Ross, Ross-Rizzo, Rotrosen,
Bogenschutz, Michael	MD	S	Psychiatry	Y	Chervinsky, Frankle, Gallagher, McGregor, Nishawala, Ross, Rotrosen
Dedania, Vaidehi	MD	S	Ophthalmology	Y	
Godina, Marina	RN	S	IRB Administration; Nursing	Y	Berkovitz, Jeschke-Lopez, Johnson
Gonen, Oded	PhD	S	Radiology	Y	Bartlett, Bencardino, Fieremans, Storey
Kadidal, Shane *	JD	NS	Legal – Prisoner Advocate	N	
Leucht, Philipp	MD, PhD	S	Orthopaedic Trauma	Y	Kirsch, Lamont, Rapp
Mehta-Lee, Shilpi	MD	S	Ob-Gyn	Y	
Panageas, Helen		NS	IRB Administration	Y	Dvorkin, Liu, McGowan, Vieira, Wallach
Ramirez, Michelle	MD	S	Pediatric Critical Care	Y	Brar, McGregpr, Oshva, Tunik
Ryan, Theresa	MD	S	Oncology	Y	Diefenbach, Esteva, Kwa, Novik, Rapp, Schiff, Wu
Saint Fleur, Shella	MD	S	Oncology	Y	Diefenbach, Esteva, Kwa, Novik, Rapp, Schiff, Wu

**Counts toward quorum only when reviewing studies subject to Subpart C*

19-AUG-2019 19-AUG-2019 19-AUG-2019 19-AUG-2019

Board E (Effective 2019-08-06)

Members; Quorum of 4; 4 for prisoner-related studies

Member	Degree(s)	Science – Non-Science	Specialty	Affiliated With NYU	Alternate(s)
More, Frederick (Chair)	DDS	S	Dentistry	Y	Donnino, Hazen, Katz, Nishawala, Novik, Wissner-Greene
Katz, Stuart (Vice-Chair)	MD	S	Cardiology	Y	Vice Chair Alternates: Donnino, Hazen, More, Nishawala, Novik, Wissner-Greene<br>Member Alternates: Culliford, Donnino
Johnson, Nadia	MS	S	IRB Administration	Y	Berkovitz, Godina, Jeschke-Lopez
Wu, Jennifer	MD	S	Oncology	Y	Diefenbach, Esteva, Kwa, Moskovits, Novik, Rapp, Ryan, Saint Fleur, Schiff
Kadidal, Shane *	JD	NS	Law-Prisoner Advocate	N	
Panageas, Helen		NS	IRB Administration	Y	Dvorkin, Liu, McGowan, Vieira, Wallach
Raskin, Joyce	JD	NS	Community Representative	Y	Abramovitz, Basu Roy, Wu

**Counts toward quorum only when reviewing studies subject to Subpart C*

19-AUG-2019

Alternates

Alternate	Degree(s)	Science – Non-Science	Specialty	Affiliated With NYU	Alternate For
Abramovitz, Rachel	LL.M., LL.B.	NS	Community Representative	N	Basu Roy, Raskin, Wu
Ballani, Kanika	PharmD	S	Pharmacy	Y	Bliss, Dubrovskaya, Wong
Barr, William	PhD	S	Neuropsychology	Y	Berk, Chevinsky, Frankle, Gallagher, Nishawala, Ross, Ross-Rizzo
Bartlett, Rachel	PhD	S	Radiology	Y	Gonen, Storey
Basu Roy, Upal	PhD, MPH	S	Community Representative	Y	Abramovitz, Raskin, Wu
Bencardino, Jenny	MD	S	Radiology	Y	Bartlett, Gonen, Schiff
Berk, Thomas	MD	S	Neurology	Y	Barr, Ross-Rizzo
Berkovitz, David	MD	S	IRB Administration	Y	Godina
Bliss, Samuel	PharmD	S	Pharmacy	Y	Ballani, Dubrovskaya, Wong
Bogenschutz, Michael	MD	S	Psychiatry	Y	Barr, Chervinsky, Frankle, Gallagher, Nishawala, Ross
Brar, Preneet	MD	S	Pediatric Endocrinology	Y	Ramirez, Wissner-Greene
Chervinsky, Alexander	PhD	S	Neuropsychology	Y	Barr, Bogenschutz, Gallagher, Nishawala, Ross, Ross-Rizzo
Culliford, Alfred	MD	S	Surgery – Cardiothoracic	Y	Donnino, Katz
DeLacure, Mark D	MD	S	Otolaryngology & Plastic Surgery	Y	Hazen
Diefenbach, Catherine	MD	S	Oncology	Y	Esteva, Kwa, Ryan, Saint Fleur, Schiff, Wu
Donnino, Robert	MD	S	Medicine – Cardiology	Y	Culliford Hazen, Katz, More, Nishawala, Novik, Wissner-Greene
Dubrovskaya, Yanina	PharmD	S	Pharmacy	Y	Ballani, Bliss, Wong
Dvorkin, Ella		NS	IRB Administration	Y	Panageas
Esteva, Francisco	MD	S	Oncology	Y	Diefenbach, Kwa, Ryan, Saint Fleur, Schiff, Wu
Faiena, Mark	PharmD	S	Pharmacy	Y	Ballani, Bliss, Dubrovskaya, Wong
Fieremans, Els	PhD		Radiology	Y	Bartlett, Gonen, Storey
Frankle, William Gordon	MD	S	Psychiatry	Y	Barr, Bogenschutz, Gallagher, Nishawala, Ross
Gallagher, Richard	PhD	S	Child & Adolescent Psychiatry	Y	Chervinsky, Frankle, Nishawala, Ross
Gonen, Oded	PhD	S	Radiology	Y	Bartlett, Storey
Hazen, Alexes	MD	S	Plastic Surgery	Y	Katz, More, Nishawala, Novik, Wissner-Greene
Jeschke-Lopez, Ikoa	MD	S	IRB Administration	Y	Godina
Johnson, Nadia	MS	S	IRB Administration	Y	Godina
Katz, Stuart	MD	S	Cardiology	Y	Donnino, Hazen, More, Nishawala, Novik, Wissner-Greene
Kim, Sunmi	MD	S	Anesthesiology	Y	Wang
Kirsch, Thorsten	MD	S	Orthopedic Surgery	Y	Lamont, Leucht, Rapp
Kwa, Maryann	MD	S	Oncology	Y	Diefenbach, Novik, Ryan, Saint Fleur, Schiff, Wu

Lamont, Justin	MD	S	Orthopedic Surgery	Y	Culliford, Hazen, Kirsch, Leucht, Rapp
Leucht, Philipp	MD, PhD	S	Orthopaedic Trauma	Y	Kirsch, Lamont, Schiff
Liu, Jasmine	BS	NS	IRB Administration	Y	Panageas
Lewis, Ariane	MD	S	Neurocritical Care	Y	Barr, Berk, Ross-Rizzo
McGowan, Richard	MLS	NS	Public Services	Y	Panageas, Vieira
McGregor, Kyle	PhD	S	Child & Adolescent Psychiatry	Y	Barr, Bogenschutz, Chervinsky,Frankle, Gallagher, Nishawala, Ross
More, Frederick	DDS	S	Dentistry – Pediatric	Y	Hazen, Katz, Nishawala, Novik, Wissner-Greene
Moskovits, Tibor	MD	S	Hematology – Oncology	Y	Diefenbach, Esteva, Kwa, Ryan, Saint Fleur, Schiff, Wu
Nishawala, Melissa	MD	S	Child & Adolescent Psychiatry	Y	Barr, Bogenschutz, Chervinsky, Frankle, Hazen, Katz, More, Novik, Ross, Wissner-Greene
Nolan, Anna	MD	S	Pulmonary	Y	
Novik, Yelena	MD	S	Oncology	Y	Diefenbach, Esteva, Katz, Kwa, Hazen, More, Nishawala, Ryan, Saint Fleur, Wissner-Greene, Wu
Oshva, Lillian	MD	S	Emergency Medicine	Y	Ramirez
Ramirez, Michelle	MD	S	Pediatric Critical Care	Y	Brar
Rapp, Timothy	MD	S	Orthopedic Surgery – Oncology	Y	Culliford, Diefenbach,Esteva, Kirsch, Kwa, Lamont, Leucht, Ryan, Saint Fleur, Schiff, Wu
Raskin, Joyce	JD	NS	Community Representative	Y	Abramovitz, Basu Roy, Wu
Ross, Stephen	MD	S	Addiction Psychiatry	Y	Barr, Bogenschutz, Chervinsky, Frankle, Nishawala
Ross-Rizzo, John	MD	S	Neurology	Y	Barr, Chervinsky,
Rotrosen, John	MD	S	Psychiatry	Y	Barr, Bogenschutz, Chervinsky, Frankle, Gallagher, Nishawala, Ross
Ryan, Theresa	MD	S	Oncology	Y	Diefenbach, Esteva, Kwa, Novik, Saint Fleur, Schiff, Wu
Saint Fleur, Shella	MD	S	Hematology - Oncology	Y	Diefenbach, Esteva, Kwa, Novik, Ryan, Schiff, Wu
Schiff, Peter	MD	S	Radiation – Oncology	Y	Diefenbach, Esteva, Kwa, Ryan, Saint Fleur, Wu
Storey, Elizabeth	PhD	S	Radiation	Y	Bartlett, Gonen
Tunik, Michael	MD	S	Pediatric EM	Y	Brar, More, Ramirez
Vieira, Dorice	MA	NS	Public Services	Y	McGowan, Panageas
Wallach, David	BA , MPH	NS	IRB Administration	Y	Panageas
Wang, Jing	MD	S	Anesthesiology	Y	Kim
Wissner-Greene, Loren	MD, MA	S	Medicine – Endocrinology – Ob-Gyn	Y	Brar, Hazen, Katz, More, Nishawala, Novik,
Wong, Doris	PharmD	S	Pharmacy	Y	Ballani, Bliss, Dubrovskaya,
Wu, Jennifer	MD	S	Oncology	Y	Diefenbach, Esteva, Kwa, Ryan, Saint Fleur
Wu, Lillian	BA, MA	NS	Community Representative	N	Abramovitz, Basu Roy, Raskin

19-AUG-2019
